# The Impact of 18 Ancestral and Horizontally-Acquired Regulatory Proteins upon the Transcriptome and sRNA Landscape of *Salmonella enterica* serovar Typhimurium

**DOI:** 10.1371/journal.pgen.1006258

**Published:** 2016-08-26

**Authors:** Aoife M. Colgan, Carsten Kröger, Médéric Diard, Wolf-Dietrich Hardt, José L. Puente, Sathesh K. Sivasankaran, Karsten Hokamp, Jay C. D. Hinton

**Affiliations:** 1 Department of Microbiology, School of Genetics and Microbiology, Moyne Institute of Preventive Medicine, Trinity College, Dublin, Ireland; 2 Institute of Microbiology, ETH Zürich, Zürich, Switzerland; 3 Departamento de Microbiología Molecular, Instituto de Biotecnología, Universidad Nacional Autónoma de Mexico, Cuernavaca, Morelos, Mexico; 4 Department of Genetics, School of Genetics and Microbiology, Smurfit Institute of Genetics, Trinity College, Dublin, Ireland; 5 Institute of Integrative Biology, University of Liverpool, Liverpool, United Kingdom; Universidad de Sevilla, SPAIN

## Abstract

We know a great deal about the genes used by the model pathogen *Salmonella enterica* serovar Typhimurium to cause disease, but less about global gene regulation. New tools for studying transcripts at the single nucleotide level now offer an unparalleled opportunity to understand the bacterial transcriptome, and expression of the small RNAs (sRNA) and coding genes responsible for the establishment of infection. Here, we define the transcriptomes of 18 mutants lacking virulence-related global regulatory systems that modulate the expression of the SPI1 and SPI2 Type 3 secretion systems of *S*. Typhimurium strain 4/74. Using infection-relevant growth conditions, we identified a total of 1257 coding genes that are controlled by one or more regulatory system, including a sub-class of genes that reflect a new level of cross-talk between SPI1 and SPI2. We directly compared the roles played by the major transcriptional regulators in the expression of sRNAs, and discovered that the RpoS (σ^38^) sigma factor modulates the expression of 23% of sRNAs, many more than other regulatory systems. The impact of the RNA chaperone Hfq upon the steady state levels of 280 sRNA transcripts is described, and we found 13 sRNAs that are co-regulated with SPI1 and SPI2 virulence genes. We report the first example of an sRNA, STnc1480, that is subject to silencing by H-NS and subsequent counter-silencing by PhoP and SlyA. The data for these 18 regulatory systems is now available to the bacterial research community in a user-friendly online resource, SalComRegulon.

## Author Summary

The transcriptional networks and the functions of small regulatory RNAs of *Salmonella enterica* serovar Typhimurium are being studied intensively. *S*. Typhimurium is becoming the ideal model pathogen for linking transcriptional and post-transcriptional gene regulation to bacterial virulence. Here, we systematically defined the regulatory factors responsible for controlling the expression of *S*. Typhimurium coding genes and sRNAs under infection-relevant growth conditions. As well as confirming published regulatory inputs for *Salmonella* pathogenicity islands, such as the positive role played by Fur in the expression of SPI1, we report, for the first time, the global impact of the FliZ, HilE and PhoB/R transcription factors and identify 124 sRNAs that belong to virulence-associated regulons. We found a subset of genes of known and unknown function that are regulated by both HilD and SsrB, highlighting the cross-talk mechanisms that control *Salmonella* virulence. An integrative analysis of the regulatory datasets revealed 5 coding genes of unknown function that may play novel roles in virulence. We hope that the SalComRegulon resource will be a dynamic database that will be constantly updated to inspire new hypothesis-driven experimentation, and will contribute to the construction of a comprehensive transcriptional network for *S*. Typhimurium.

## Introduction

*Salmonella enterica* serovar Typhimurium (*S*. Typhimurium) is an important foodborne pathogen that causes self-limiting gastroenteritis, or more serious systemic infections in susceptible hosts. In the developed world, there are an estimated 93.8 million incidences of salmonellosis caused by non-typhoidal *Salmonella* (NTS) strains, resulting in 155,000 deaths each year [1]. In developing countries, NTS strains cause bloodstream infections that kill about 20% of patients. This high mortality rate reflects the combination of pre-disposing conditions such as HIV, malaria and malnutrition, and the emergence of invasive NTS strains [2, 3]. *S*. Typhimurium colonises a wide range of mammals and birds, and encounters a series of stressful conditions within various host environments. The bacteria express a Type III Secretion System (T3SS) encoded on a pathogenicity island (SPI1) that mediates invasion of the host intestinal epithelium. Once internalised, *S*. Typhimurium expresses a second T3SS, encoded on a second pathogenicity island (SPI2), which is responsible for its survival and replication in the intracellular environment within the *Salmonella* containing vacuole (SCV) and for the establishment of systemic infection [4, 5].

*Salmonella* has the ability to rapidly remodel its gene expression profile when exposed to different spatial and temporal cues. This co-ordinated transcriptional programme allows *S*. Typhimurium to interact with the microbiota and the mammalian host, to multiply and survive in the host intestine and to cause systemic disease [6]. For *Salmonella* bacteria, infection involves the generation of genetically-identical cells with different but cooperating phenotypes to ensure fitness and optimal spatio-temporal gene expression [7–12]. RNA-seq analysis of individual host cells has revealed that the gene expression profile of infecting bacterial cells can have profound effects on the resulting host response [13]. Such a flexible bacterial gene expression program must be tightly controlled by the dynamic interactions of hundreds of transcriptional regulators. *S*. Typhimurium encodes 156 protein factors which act at the level of the initiation of transcription [14]. In addition, gene expression is regulated at the post-transcriptional level, largely by *trans*-acting small regulatory RNAs (sRNA) [15]. *Trans*-acting sRNAs usually affect expression of target genes through short imperfect base-pairing interactions, and often require the RNA chaperone, Hfq [16].

We require an integrated understanding of the regulatory inputs that coordinate the expression of all RNA transcripts of *Salmonella*. Here, we explore the interconnections between transcriptional and post-transcriptional regulation of *S*. Typhimurium protein coding genes and sRNAs using bacterial mutants that lack key components of the global transcriptional networks that are associated with SPI1 and SPI2 expression. We used an RNA-seq-based transcriptomic approach to explore the regulons of 18 virulence-associated regulatory systems including sigma factors, transcription factors, two-component systems and an RNA chaperone, under *in vitro* conditions.

Our particular focus was the regulation of sRNA gene expression and we identified 124 sRNA genes that are controlled by at least one of the regulatory systems tested in this work. Molecular and computational approaches were used to validate regulatory interactions. We showed that the putative virulence-associated sRNA, STnc1480, is silenced by the nucleoid-associated protein H-NS, and that the transcription factors PhoP and SlyA counteract the repressive effects of H-NS at the STnc1480 promoter. Finally, we describe 13 sRNAs which we predict to play important roles in *S*. Typhimurium virulence, based on their patterns of regulation.

We assembled an online compendium of our RNA-seq-based transcriptomic analysis of the regulons of 18 systems that control *S*. Typhimurium virulence, as a community resource. One important caveat is that these regulons contain genes that are both directly and indirectly regulated. Further experiments will be required to identify the binding sites for each transcription factor across the chromosome. This investigation of the regulatory inputs to the expression of *S*. Typhimurium coding genes and sRNAs significantly extends current knowledge about the interconnections between transcriptional and post-transcriptional regulatory elements, and is a step towards the elucidation of the topology of regulatory networks that control *S*. Typhimurium pathogenesis.

## Results and Discussion

### RNA-seq analysis of regulatory mutants of *S*. Typhimurium under infection-relevant growth conditions

Previously, we profiled the transcriptome of wild-type *S*. Typhimurium 4/74 under 22 environmental conditions [9]. We discovered that *S*. Typhimurium possesses 280 sRNAs and that the expression of each sRNA was highly dynamic and environmentally-responsive [9]. However, little was known about the protein factors responsible for modulating sRNA expression. Here we investigated global transcriptional and post-transcriptional regulators of *Salmonella* gene expression to identify regulatory inputs that control the dynamic expression of *S*. Typhimurium sRNAs.

We selected a panel of 18 virulence-associated *S*. Typhimurium regulatory proteins, and disrupted their expression by generating a set of isogenic deletion mutants (Tables 1 and 2; Materials and Methods). Our published transcriptomic and transcriptional start site (TSS) data from wild-type strain 4/74 [9] were used to investigate whether the expression of downstream genes was affected by the genetic constructions used to delete the individual regulatory genes. Typically, the entire coding regions of 18 regulatory genes were removed. To avoid polar effects, resistance cassettes were removed from mutant strains if the deleted gene had its own TSS and was in the 5’ region of an operon. For the *sirA* mutation, a 432 bp region at the 5’ end of the *sirA* coding sequence (CDS) was deleted to maintain an intact *uvrC* TSS. For the *slyA* mutation, the 5’ and 3’ ends of the *slyA* CDS were left intact to avoid disruption of *anmK* expression and deletion of the overlapping 3’ end of the *slyB* CDS on the opposite strand.

**Table 1:** Bacterial strains and plasmids.

Name	Genotype	Reference
**Bacterial strains**		
	Wild-type prototroph *S*. Typhimurium 4/74. Str^R^	[88]
JH3630	*S*. Typhimurium 4/74 Δ*rpoE*::*frt*	P22 transduction from JVS-1028
JH3632	*S*. Typhimurium 4/74 Δ*fur*::*frt*	P22 transduction from JH3305
JH3635	*S*. Typhimurium 4/74 Δ*hilD*::*frt*	P22 transduction from JVS-5924
JH3636	*S*. Typhimurium 4/74 Δ*slyA*::*kan*	This Study
JH3637	*S*. Typhimurium 4/74 Δ*phoB/R*::*kan*	P22 transduction from JH3312
JH3643	*S*. Typhimurium 4/74 Δ*fliZ*::*frt*	This Study
JH3652	*S*. Typhimurium 4/74 Δ*ompR/envZ*::*frt*	P22 transduction from [99]
JH3653	*S*. Typhimurium 4/74 Δ*dam*::*frt*	P22 transduction from [37]
JH3657	*S*. Typhimurium 4/74 Δ*ssrA/B*::*frt*	This Study
JH3675	*S*. Typhimurium 4/74 Δ*ssrA*::*frt*	This Study
JH3733	*S*. Typhimurium 4/74 Δ*ssrB*::*frt*	This Study
JH3660	*S*. Typhimurium 4/74 Δ*phoP/Q*::*frt*	This Study
JH3763	*S*. Typhimurium 4/74 Δ*phoP*::*frt*	P22 transduction from JVS-8781
JH3673	*S*. Typhimurium 4/74 Δ*barA/sirA-432*::*kan*	This Study
JH3674	*S*. Typhimurium 4/74 Δ*rpoS*::*kan*	P22 transduction from JH3575
JH3584	*S*. Typhimurium 4/74 Δ*hfq*::*kan*	P22 transduction from JVS-00225
JH3765	*S*. Typhimurium 4/74 Δ*hilA*::*frt*	P22 transduction from JVS-1195
JH3766	*S*. Typhimurium 4/74 Δ*hilC*::*cat*	
JH3767	*S*. Typhimurium 4/74 Δ*hilE*::*cat*	P22 transduction from M2008 [11]
JH3774	*S*. Typhimurium 4/74 *hns*-1::*kan*	[100]
JH3775	*S*. Typhimurium 4/74 *phoP*-3xFLAG (C-terminal)	This Study
JH3777	*S*. Typhimurium 4/74 *hns*-3xFLAG::*kan* (C-terminal)	[71]
JH3782	*S*. Typhimurium 4/74 Δ*slyA hns*-1::*kan*	This Study
JH3783	*S*. Typhimurium 4/74 Δ*phoP hns*-1::*kan*	This Study
TOP10	*E*. *coli* TOP10 F- mcrA Δ(mrr-hsdRMS-mcrBC) φ80lacZΔM15 ΔlacX74 nupG recA1 araD139 Δ(ara-leu)7697 galE15 galK16 rpsL(StrR) endA1 λ-	Invitrogen
**Plasmids**		
pKD3	Template plasmid for gene deletion; Amp^R^, FRT-flanked *cat* gene	[89]
pKD4	Template plasmid for gene deletion; Amp^R^, FRT-flanked *kan* gene	[89]
pKD46	Red helper plasmid; repA101 (ts), pBAD-*γ-β-exo*, *araC*^+^, Amp^R^	[89]
pCP20	Plasmid for temperature sensitive FLP synthesis; ts-rep, *FLP*, Cm^R^, Amp^R^	[91]
pSUB11	Template plasmid for epitope tagging; Amp^R^, FRT-flanked 3xFLAG tag and *kan* gene	[90]
pBAD-myc-hisA	pBR322 origin, araC^+^, araBAD, C-terminal myc and polyhistidine tags, Amp^R^	Invitrogen
pBAD-*slyA*	*slyA* gene cloned in MCS of pBAD-myc-hisA	This Study
pBAD-*phoP*	*phoP* gene cloned in MCS of pBAD-myc-hisA	This Study

Antibiotics were used at the following final concentrations: Ampicillin (Amp) 100 μg/mL, Kanamycin (Kan) 50 μg/mL, Chloramphenicol (Cm) 35 μg/mL.

**Table 2:** Panel of regulatory mutants and environmental conditions used for RNA-seq experiments.

Strain	Function of Regulator	Growth Condition	Description of Growth	Rationale for Chosen Growth Condition
wild-type		Mid exponential phase (MEP)	growth to OD_600_0.3 in Lennox broth	Exponentially growing cultures contain hemi-methylated DNA sites for targeting by the Dam methylase.
Δ*dam*	DNA adenine methyltransferase protein. Targets GATC sites on hemi-methylated DNA, important for initiation of chromosome replication and mismatch DNA repair [101]. Dam activates SPI1 genes through post-transcriptional control of HilD [38].	Mid exponential phase (MEP)	growth to OD_600_0.3 in Lennox broth	
wild-type		Intermediate exponential phase (IEP)	growth to OD_600_0.8 in Lennox broth	*S*. Typhimurium grown to OD_600_0.8 in Lennox broth expresses SPI1 in a bistable fashion and the competitive growth disadvantage between cells expressing SPI1 and their non-SPI1-expressing siblings is greatest at this time point [11].
Δ*hilD*	SPI1-encoded HilD is the primary regulator of genes necessary for the expression of the SPI1 T3SS, through formation of a feed-forward regulatory loop with HilC and RtsA [102].	Intermediate exponential phase (IEP)	growth to OD_600_0.8 in Lennox broth	
Δ*hilC*	SPI1-encoded HilC forms part of a feed-forward regulatory loop necessary for the expression of the SPI1 T3SS [102].	Intermediate exponential phase (IEP)	growth to OD_600_0.8 in Lennox broth	
Δ*hilA*	SPI1-encoded HilA is the central regulator of SPI1 T3SS genes, either directly or indirectly through control of another SPI1-encoded TF, InvF [103].	Intermediate exponential phase (IEP)	growth to OD_600_0.8 in Lennox broth	
Δ*hilE*	The *hilE* gene is transcriptionally activated by the regulator of fimbrial gene expression, FimZ [29], and HilE interacts with the HilD protein to repress *hilA* transcription [52].	Intermediate exponential phase (IEP)	growth to OD_600_0.8 in Lennox broth	
wild-type		Early stationary phase (ESP)	growth to OD_600_2.0 in Lennox broth	We have previously shown that growth to ESP induces expression of genes encoding the components of the SPI1 T3SS [9].
Δ*fur*	Fur is the ferric uptake regulator, responsible for maintaining cellular iron homeostasis. In iron-replete conditions Fe^2+^-bound Fur binds to promoters of target genes, involved in acquisition and transport of iron, and blocks their transcription. When iron levels are low, Fur dissociates from the ferrous ion and the target DNA, allowing target gene transcription [104]. Fur activates *hilA* expression through transcriptional repression of the *hns* gene. H-NS normally silences *hilA* expression and is counter-silenced by HilD at the *hilA* promoter [20].	Early stationary phase (ESP)	growth to 0D_600_2.0 in Lennox broth	
Δ*hilD*	SPI1-encoded HilD is the primary regulator of all genes necessary for the expression of the SPI1 T3SS, through formation of a feed-forward regulatory loop with HilC and RtsA [102].	Early stationary phase (ESP)	growth to OD_600_2.0 in Lennox broth	
Δ*barA/sirA*	BarA/SirA is a TCS involved in the indirect activation of SPI1 via the sRNAs CsrB and CsrC. CsrB and CsrC bind and titrate the RNA-binding protein CsrA from its target mRNAs, including *hilD* mRNA [18, 105, 106].	Early stationary phase (ESP)	growth to OD_600_2.0 in Lennox broth	
Δ*fliZ*	FliZ is involved in regulation of flagellar gene expression. FliZ also activates *hilA* expression through post-translational interaction with the HilD protein [22].	Early stationary phase (ESP)	growth to OD_600_2.0 in Lennox broth	
Δ*hfq*	Hfq is an RNA binding protein that is a core component of post-transcriptional regulatory networks, through facilitation and stabilisation of interactions between *trans*-acting sRNAs and their target mRNAs [77]. Hfq affects gene function in a number of ways and a Δ*hfq* mutant is attenuated for invasion, intracellular replication and motility [107].	Early stationary phase (ESP)	growth to OD_600_2.0 in Lennox broth	
wild-type		Late stationary phase (LSP)	growth to OD_600_2.0 in Lennox broth + 6 hours further growth	Growth to late stationary phase exposes *S*. Typhimurium to nutrient deprivation, oxygen depletion and membrane damage. This environment activates the general stress response sigma factor, RpoS, and the alternative sigma factor RpoE, associated with the extracytoplasmic stress response.
Δ*rpoE*	RpoE is an alternative sigma factor that regulates genes encoding components of the extracytoplasmic stress response (ESR). RpoE contributes to *S*. Typhimurium pathogenesis by regulating expression of genes required for survival of stressful host environments and a Δ*rpoE* mutant is attenuated for virulence in macrophages and mice [34].	Late stationary phase (LSP)	growth to OD_600_2.0 in Lennox broth + 6 hours further growth	
Δ*rpoS*	RpoS is the general stress response alternative sigma factor, responsible for transcription of genes necessary for bacterial survival in a diverse range of stressful conditions. RpoS contributes to *S*. Typhimurium pathogenesis through activation of the virulence plasmid-encoded *spv* genes and curli fibre-encoding *csg* genes [32, 108, 109]. A Δ*rpoS* mutant is attenuated for virulence following oral infection of mice [32].	Late stationary phase (LSP)	growth to OD_600_2.0 in Lennox broth + 6 hours further growth	
wild-type		SPI2-inducing (InSPI2)	growth to OD_600_0.3 in PCN (pH 5.8, 1 mM MgCl^2+^, 0.4 mM P_i_)	It has previously been shown that a slightly acidic pH (5.8) and a limitation of inorganic phosphate (0.4 mM) induces expression of the SPI2 T3SS, as these conditions mimic aspects of the conditions encountered by intra-macrophage *Salmonella* [31].
Δ*phoB/R*	PhoBR comprise a TCS that responds to environmental phosphate levels and regulates target genes to maintain cellular phosphate homeostasis. PhoB/R is thought to play a regulatory role in the phosphate-limiting intracellular environment. PhoB/R is involved in repression of *hilA* expression when phosphate levels are low and invasion gene expression is no longer required [103].	SPI2-inducing (InSPI2)	growth to OD_600_0.3 in PCN (pH 5.8, 1 mM MgCl^2+^, 0.4 mM P_i_)	
Δ*slyA*	SlyA is required for resistance to oxidative stress and antimicrobial peptides, macrophage survival and virulence in mice but is not required for invasion of epithelial cells. SlyA binds to and activates transcription from the *ssrA* promoter of the SPI2-encoded TCS SsrA/B [44, 110]. SlyA plays a key role in regulation of virulence gene expression through counter-silencing of H-NS target genes [111].	SPI2-inducing (InSPI2)	growth to OD_600_0.3 in PCN (pH 5.8, 1 mM MgCl^2+^, 0.4 mM P_i_)	
Δ*ompR/envZ*	OmpR/EnvZ is a TCS involved in the acid tolerance response in *S*. Typhimurium. OmpR binds to and directly regulates expression from the *ssrA* promoter [99], while, in conjunction with the nucleoid-associated protein Fis, OmpR primes SPI2 genes for expression while *S*. Typhimurium is still in the host intestinal lumen [112].	SPI2-inducing (InSPI2)	growth to OD_600_0.3 in PCN (pH 5.8, 1 mM MgCl^2+^, 0.4 mM P_i_)	
Δ*phoP/Q*	PhoP/Q is a TCS that senses the concentration of divalent cations, such as Mg^2+^ and Ca^2+^ and activates target genes in response to starvation of these cations, for example during intracellular growth. PhoP/Q contributes to *S*. Typhimurium pathogenesis through differential control of SPI1 and SPI2 gene expression. PhoP negatively regulates *hilA*, via activation of *hilE* in a FimZ-dependent manner [29], and positively regulates SPI2 expression through transcriptional activation of *ssrB* and post-transcriptional activation of *ssrA* [113].	SPI2-inducing (InSPI2)	growth to OD_600_0.3 in PCN (pH 5.8, 1 mM MgCl^2+^, 0.4 mM P_i_)	
Δ*ssrA*	SsrA is the SPI2-encoded sensor kinase of the SsrA/B TCS that is the central regulator of all components of the SPI2 T3SS [56].	SPI2-inducing (InSPI2)	growth to OD_600_0.3 in PCN (pH 5.8, 1 mM MgCl^2+^, 0.4 mM P_i_)	
Δ*ssrB*	SsrB is the SPI2-encoded response regulator of the SsrA/B TCS that is the central regulator of all components of the SPI2 T3SS [56].	SPI2-inducing (InSPI2)	growth to OD_600_0.3 in PCN (pH 5.8, 1 mM MgCl^2+^, 0.4 mM P_i_)	
Δ*ssrA/B*	The SsrA/B TCS is the central regulator of all components of the SPI2 T3SS. Multiple environmental and regulatory inputs are integrated at the *ssrA* and *ssrB* promoters to ensure *Salmonella* survival within the intracellular environment [56].	SPI2-inducing (InSPI2)	growth to OD_600_0.3 in PCN (pH 5.8, 1 mM MgCl^2+^, 0.4 mM P_i_)	
Δ*hilD*	HilD is the primary regulator of all components of the SPI1 T3SS and also mediates regulatory cross-talk between SPI1 and SPI2, resulting in the activation of the SPI2 T3SS through counter-silencing of H-NS at the *ssrB* promoter [30].	SPI2-inducing (InSPI2)	growth to OD_600_0.3 in PCN (pH 5.8, 1 mM MgCl^2+^, 0.4 mM P_i_)	

**Fig 1:**
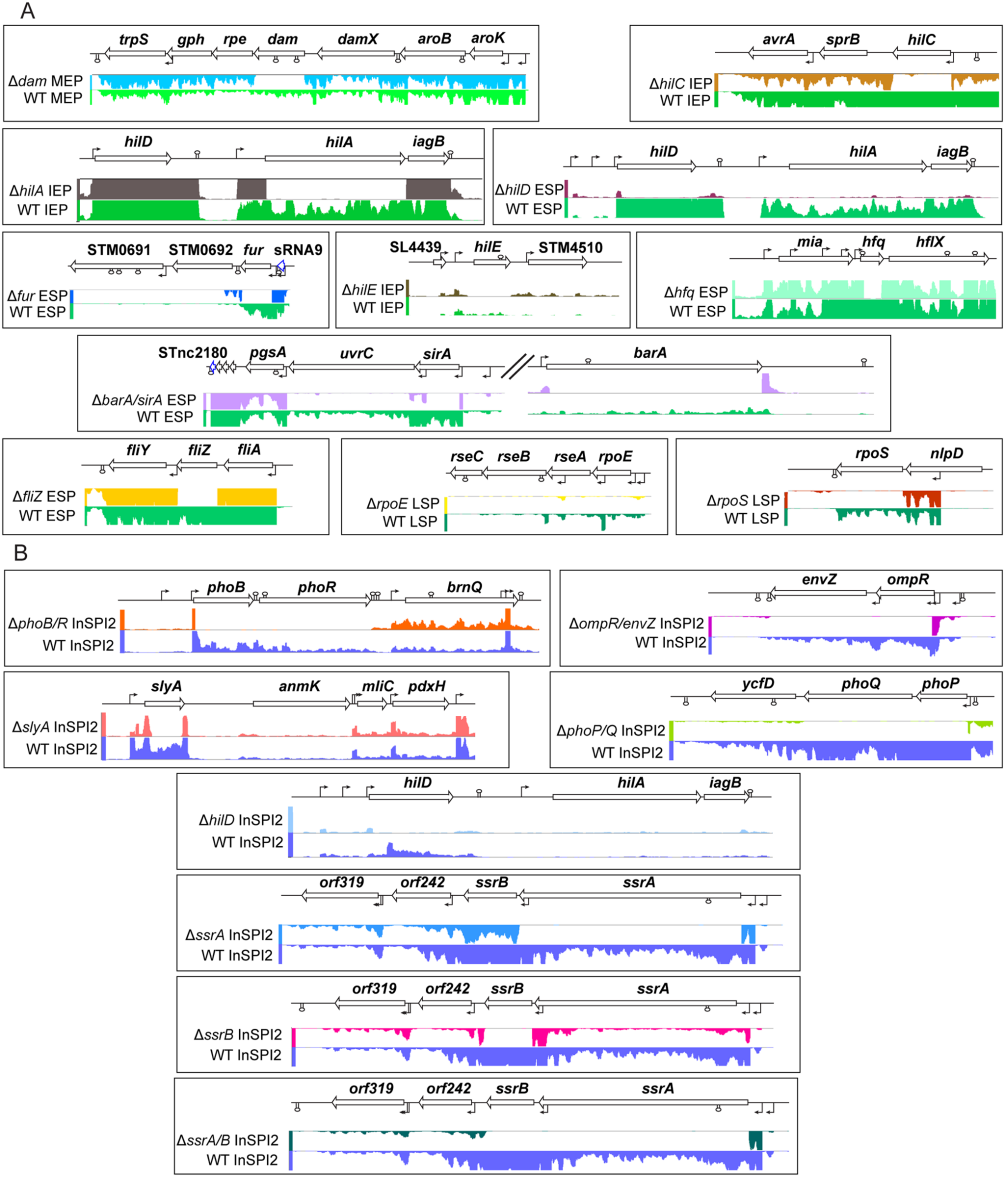
Confirmation of 18 chromosomal deletions by RNA-seq. Visualisation of sequence reads in the surrounding region of each deleted gene in the relevant mutant strain and wild-type comparator, grown under identical conditions in Lennox medium (A) or SPI2-inducing PCN medium (B), in the Integrated Genome Browser. The colours of each track represent the sequencing reads which map to that locus and the height of the normalised reads is directly proportional to the level of expression at that locus (Materials and Methods). The individual panels demonstrate that the gene encoding each regulator was expressed in the wild-type strain under the growth condition chosen for analysis of that regulator, and that no sequencing reads mapped to the deleted region of the mutant strain. Neighbouring genes were generally not affected by polar mutations (see S1 Text for exceptions). White arrows with a black outline denote protein-coding genes. White arrows with a blue outline denote sRNA genes. Black bent arrows indicate TSS. All arrows indicate the direction of transcription. Predicted Rho (ρ)-independent terminators [93] are denoted by stem-loop structures.

It is evident from Fig 1A and 1B that each of the 18 selected regulatory genes was expressed in the wild-type strain under the growth conditions used in this study, and that the gene deletions did not usually cause significant polar downstream effects. Exceptions are discussed in S1 Text. Analysis of the transcriptome and sRNA expression landscape in this panel of mutants, using RNA-seq, revealed the complexity of gene regulation, and offers clues to the function of uncharacterised sRNAs.

The panel of regulatory proteins (Table 2) included SPI1-encoded transcription factors (HilA, HilC and HilD) and transcription factors previously reported to control SPI1 gene expression (HilE, BarA/SirA, Fur and FliZ) [17–24]. Mutants lacking these transcription factors were studied under SPI1-inducing conditions. The SPI2-encoded regulators (SsrA/B) and regulatory systems that play an important role in intracellular survival and replication of *S*. Typhimurium (SlyA, HilD, OmpR/EnvZ, PhoP/Q, PhoB/R) [25–30] were investigated during growth in SPI2-inducing conditions [31]. Additionally, the regulons of the DNA adenine methyltransferase protein, Dam, and two alternative sigma factors, RpoS (σ^38^) and RpoE (σ^24^), were investigated in Lennox medium; these three transcription factors (TF) also modulate *S*. Typhimurium virulence gene expression [32–38]. Some of the regulatory proteins are encoded by the core ancestral *S*. Typhimurium genome, while others are associated with horizontally-acquired pathogenicity islands (S5 Table). The selected regulators represent a diverse range of systems that control the extracellular (SPI1-dependent) and intracellular (SPI2-dependent) infection modes of *S*. Typhimurium (S1 Fig), as recently reviewed [39].

The regulatory role of each protein was determined by comparing the transcriptome of isogenic mutant and wild-type strains grown in identical environmental conditions. The rationale and abbreviation used for each growth condition is detailed in Table 2. The wild-type strain and the regulatory mutants shared a similar growth rate in the relevant media (S1 Table).

The mapping statistics from three independent RNA-seq runs are detailed in S2 Table. The genes controlled by each of the 18 regulatory systems were defined, as described in Materials and Methods. S3 Table contains lists of all differentially-expressed protein-coding (CDS) genes and sRNA genes (>3-fold change in expression in mutant strain compared to wild-type) in the panel of regulatory mutants. As previously mentioned, these regulons contain genes that are both directly and indirectly regulated, and further experiments will be required to identify the binding sites for each TF across the chromosome.

### Control of SPI1 and SPI2 expression by transcriptional regulatory systems under infection-relevant *in vitro* conditions

**Fig 2:**
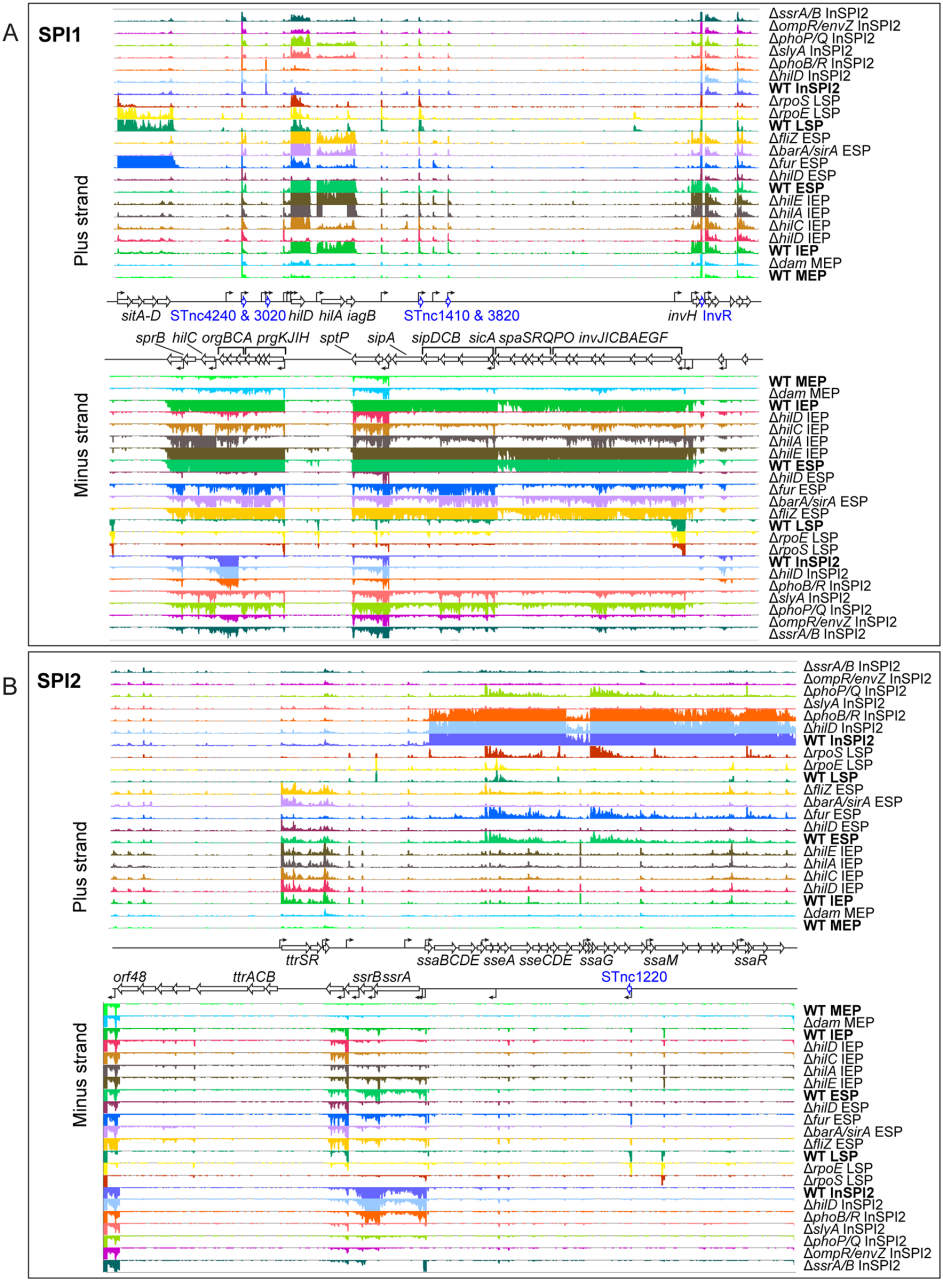
Visualisation of mapped sequence reads in the SPI1 (A) and SPI2 (B) pathogenicity islands in wild-type *S*. Typhimurium and the panel of regulatory mutants. Strains were grown under 5 *in vitro* environmental conditions, as indicated. Abbreviations for the 5 growth conditions (MEP, IEP, ESP, LSP and InSPI2) are defined in Materials and Methods and Table 2. CDS names are labelled in black, small RNA gene names in blue. All arrows indicate the direction of transcription, and transcriptional start sites are indicated as bent arrows. Strain names and conditions are labelled at the right of the image and the wild-type comparators for the relevant mutants are labelled in bold. The figure was prepared with the IGB browser, and the scale is 0–100 normalized reads for every sample.

As SPI1 and SPI2 are the two main *Salmonella* pathogenicity islands, we began by investigating the transcriptional landscape of these regions in our panel of regulatory mutants (Fig 2A and 2B). To simplify analysis of the SsrA/B regulon, differentially-expressed genes in the Δ*ssrA*, Δ*ssrB* and Δ*ssrAB* mutant strains were combined as Δ*ssrAB* (see S2 Fig and S3 Table for individual Δ*ssrA* and Δ*ssrB* datasets). Expression of the SPI1 CDS genes was reduced by an average of 40-fold in the absence of the SPI1-encoded transcription factors. The SPI2 CDS gene expression was reduced by an average of 109-fold in the absence of the SPI2-encoded and SPI2-associated transcription factors.

**Fig 3:**
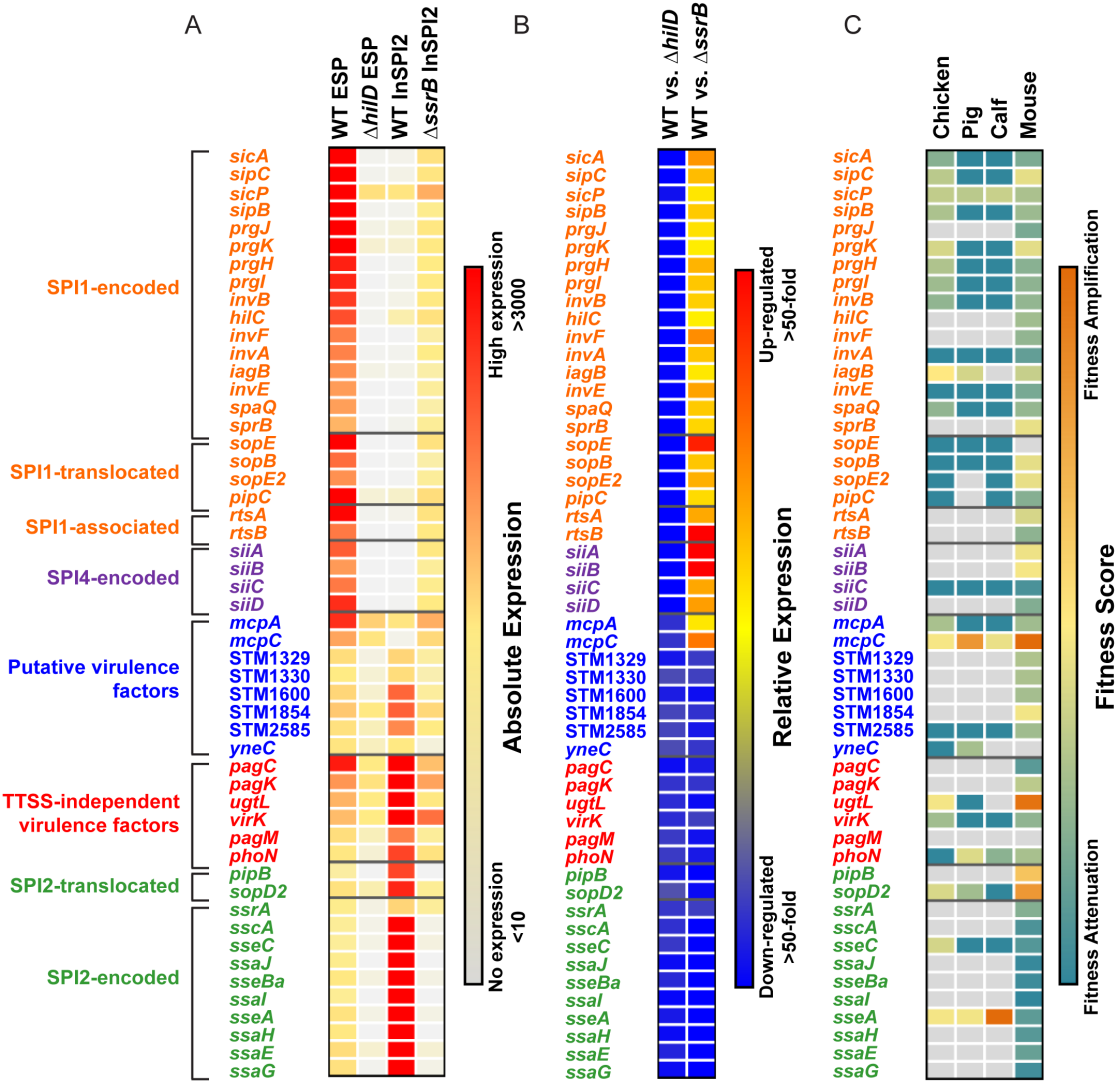
Overlap between HilD and SsrB-controlled gene expression reveals 8 putative virulence factors. Absolute expression (A) and relative expression (B) of the 52 CDS genes which are differentially-expressed (>3-fold) in the absence of HilD and SsrB, compared to wild-type grown under ESP and InSPI2 conditions, respectively (putative co-regulated genes). (C) Presence of a transposon insertion in the 52 co-regulated genes leads to attenuation or amplification of fitness in chicken, pig or calf models [41]. The scoring methods used by the authors of each publication were applied independently to each dataset (Materials and Methods). A negative score indicates attenuation of fitness as a result of the transposon insertion (blue), while an increase in fitness is denoted by a positive fitness score (orange). If no output reads were identified for a particular insertion an arbitrary negative fitness score of -15 was assigned [41]. Replacement of co-regulated genes with either a sense-oriented Kanamycin resistance cassette or an antisense-oriented chloramphenicol resistance cassette resulted in fitness attenuation in colonisation of either the spleen or liver of BALB/c mice following infection via the i.p. route [42]. A negative fitness score indicates attenuation of fitness as a result of the gene deletion (blue), while an amplification of fitness is reflected by a positive fitness score (orange). Grey indicates that no virulence data were available for that gene. Eight genes that were co-regulated by HilD and SsrB have putative roles in virulence, and are indicated in blue.

Analysis of gene expression across a panel of regulatory mutants, rather than focusing on individual mutants in isolation, allowed us to observe patterns of co-regulation between *Salmonella* regulons. Our transcriptomic approach highlights the regulatory connections that exist between the two main pathogenicity islands, and shows the HilD-mediated cross-talk between SPI1 and SPI2 that has previously been reported [30]. We also found CDS genes that were differentially-expressed (>3-fold) in the absence of both HilD and SsrB, and the majority of these 52 HilD/ SsrB-controlled genes were virulence-associated (Fig 3A and 3B). During growth at ESP, the absence of HilD caused the down-regulation of many SPI2-encoded and SPI2-associated genes which confirmed the previously reported HilD-mediated cross-talk between SPI1 and SPI2. Mechanistically, HilD indirectly regulates SsrB-regulated genes by antagonizing the H-NS-mediated repression of *ssrAB* at stationary phase under SPI-1 inducing conditions [30] (see also Fig 2). However, the up-regulation of SPI1-associated genes in the absence of SsrB suggests an additional layer of transcriptional control for SPI1 genes, via SsrB-mediated repression under SPI2-inducing conditions.

Eight of the 52 HilD/ SsrB-controlled genes have not previously been directly linked to the SPI1 or SPI2 regulons and in most cases have no known function (Function UNknown, FUN). Two of the 8 genes, *mcpA* (STM3138) and *mcpC* (STM3216), are predicted to encode *Salmonella*-specific methyl-accepting chemotaxis proteins [40] and display similar expression patterns to the SPI1-encoded, SPI1-associated and SPI4-encoded genes which are directly or indirectly activated by HilD, and repressed by SsrB, but are not regulated by HilA suggesting they are controlled by the HilD feed-forward loop. The other six genes STM1329, STM1330, STM1600, STM1854, STM2585 and *yneC* (STM4079S) show a pattern of expression that resembles the SPI2-encoded and SPI2-associated genes, that are positively regulated by both HilD and SsrB under SPI1 and SPI2-inducing conditions, respectively, and are upregulated within macrophages [14].

We speculate that the HilD/ SsrB-controlled FUN genes could play a role in virulence and to further support this hypothesis, we interrogated published data from transposon-directed insertion site sequencing (TraDIS) involving oral infections of chicken, pigs and calves [41] and data from intraperitoneal infection of BALB/c mice using single gene deletion mutant libraries [42]. The importance of the 52 HilD/ SsrB-controlled genes during infection of these 4 animal models is summarised in Fig 3C.

Seven of the 8 HilD/ SsrB-controlled FUN genes are required for infection of at least one of the animal models. The Gifsy-1-encoded putative transposase gene, STM2585, is required for virulence in all 4 animal models. Moreover, a deletion mutant of STM2585 (*steE*) is attenuated for colonization of mouse spleen and the SteE protein is translocated into the host cell cytoplasm [43]. Furthermore, STM2585 and STM1330 are regulated by SlyA and PhoP [44], the master regulators of the SsrB regulon. In contrast, *mcpC* mutants show increased fitness in all animal models, consistent with a role in down-regulating the expression or function of specific traits that may have a fitness cost for the bacterium during the invasion process.

The identification of these putative novel virulence factors through investigation of their regulatory patterns highlights the power of our approach to both recapitulate current knowledge on SPI1 and SPI2 regulatory cross-talk, and to predict the role of novel FUN genes in virulence.

### The influence of transcriptional regulatory systems upon the expression landscape of sRNAs

The CDS targets of many *Salmonella* TFs have previously been identified by microarray and single gene analysis studies [37, 44–50], and data from these published studies were used to validate our method of investigating bacterial regulons. Table 3 highlights the effects of the panel of regulatory mutants on flagellar, SPI1 and SPI2 gene expression in the context of the literature. Our data confirm published *S*. Typhimurium virulence gene regulatory interactions, and identify new regulatory links described below. Any differences between the regulons defined in this study and the literature are discussed in S1 Text, and typically arise from differences in the growth conditions that were used, strain background or the increased dynamic range and sensitivity of the RNA-seq-based method of transcriptional profiling. Overall, these comparisons confirm that RNA-seq-based transcriptomics accurately characterises CDS gene expression, and so allows individual sRNAs to be assigned to specific cellular regulons.

**Table 3:** Comparison of virulence gene expression in published regulons with panel of regulatory mutants^a^.

	Flagella	SPI1	SPI2	Supported by the literature	Disagrees with the literature	Novel findings	Strain
**HilA**				HilA does not affect *flhD* expression [114]. HilA activates SPI1 [115].			14028 [114] SL1344 [115]
**HilC**				HilC activates SPI1 [116].			SL1344 [116]
**HilD**				HilD activates *flhD* expression [114]. HilD activates SPI1 [102]. HilD activates SPI2 [30].			14028 [114] 14028[102] SL1344 [30]
**BarA/SirA**				BarA/SirA represses flagellar gene expression [17]. BarA/SirA activates SPI1 [18].	*fljB* expression decreases in the absence of BarA/SirA.	Expression of SPI2 genes decreases in the absence of BarA/SirA.	14028 [17] 14028 [18]
**FliZ**				FliZ activates most flagellar genes [117]. FliZ activates SPI1 [118].		Expression of *fljAB* increases in the absence of FliZ. Expression of SPI2 genes decreases in the absence of FliZ.	14028 [117] SL1344 [118]
**Fur**				Fur activates flagellar genes [119]. Fur activates SPI1 genes [19]. Fur represses SPI2 genes [120].			14028 [119] 14028 [19] 14028 [120]
**Hfq**				Hfq activates flagellar genes, SPI1 genes and SPI2 genes [78].			SL1344 [78]
**HilE**							
**Dam**				Dam methylation represses *fliD* [37].	Expression of *fliC* decreases in the absence of Dam. Expression of SPI1 increases in the absence of Dam.	Expression of *fljAB* increases in the absence of Dam.	SL1344 [37]
**RpoE**							
**RpoS**				*fliC* expression decreases in the absence of RpoS [63].		Expression of *invF* decreases in the absence of RpoS. Expression of *ssa* genes increase in the absence of RpoS.	14028 [63]
**PhoB/R**							
**PhoP/Q**				PhoP/Q represses flagellar genes [121]. PhoP/Q represses SPI1 [29]. PhoP/Q activates SPI2 [113].			SL1344 [121] SL1344 [29] 14028 [113]
**OmpR/EnvZ**				OmpR/EnvZ represses flagellar genes [50]. OmpR/EnvZ represses SPI1 [57]. OmpR/EnvZ activates SPI2 [26].			SL1344 [50] SL1344 [57] 14028 [26]
**SlyA**				SlyA activates SPI2 [44].		Expression of flagellar genes increases in the absence of SlyA. Expression of SPI1 genes increases in the absence of SlyA.	14028 [44]
**SsrA/B**				SsrA/B activates SPI2 [25].		Expression of flagellar genes increases in the absence of SsrA/B. Expression of SPI1 genes increases in the absence of SsrA/B.	SL1344 [25]

^a^ Coloured squares indicate the putative regulatory effect of the indicated regulatory system on the indicated group of genes based on differential gene expression in the panel of regulatory mutants in this study. Blue indicates putative repression (higher gene expression in mutant than wild-type); Red indicates putative activation (lower gene expression in mutant than wild-type); Magenta indicates a mixture of up- and down-regulated genes; Grey indicates no change in expression.

**Fig 4:**
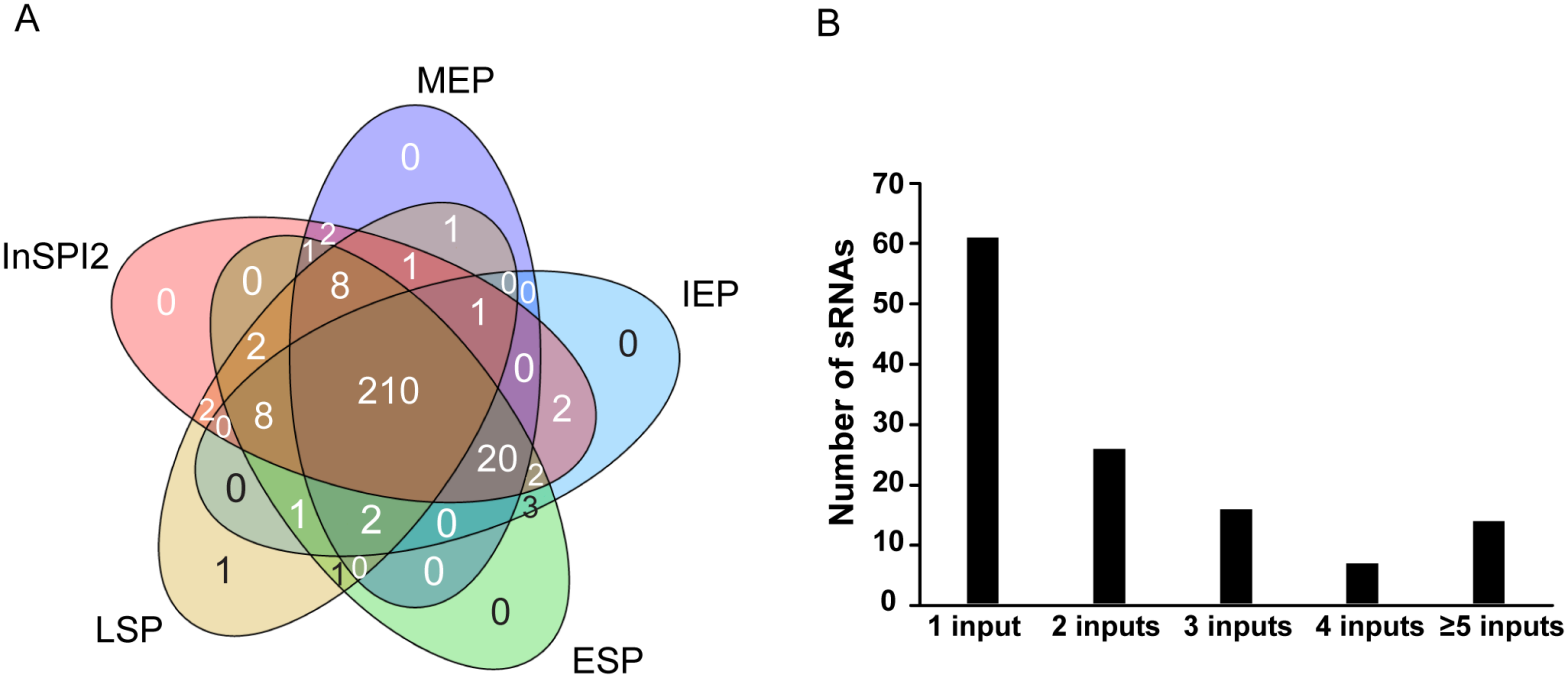
Expression and regulation of *S*. Typhimurium sRNAs. (A) sRNAs expressed in wild-type *S*. Typhimurium 4/74 under 5 *in vitro* environmental conditions. (B) Bar chart showing the number of differentially-expressed sRNAs in the context of the number of putative regulatory inputs to each gene from the panel of 18 TFs.

**Fig 5:**
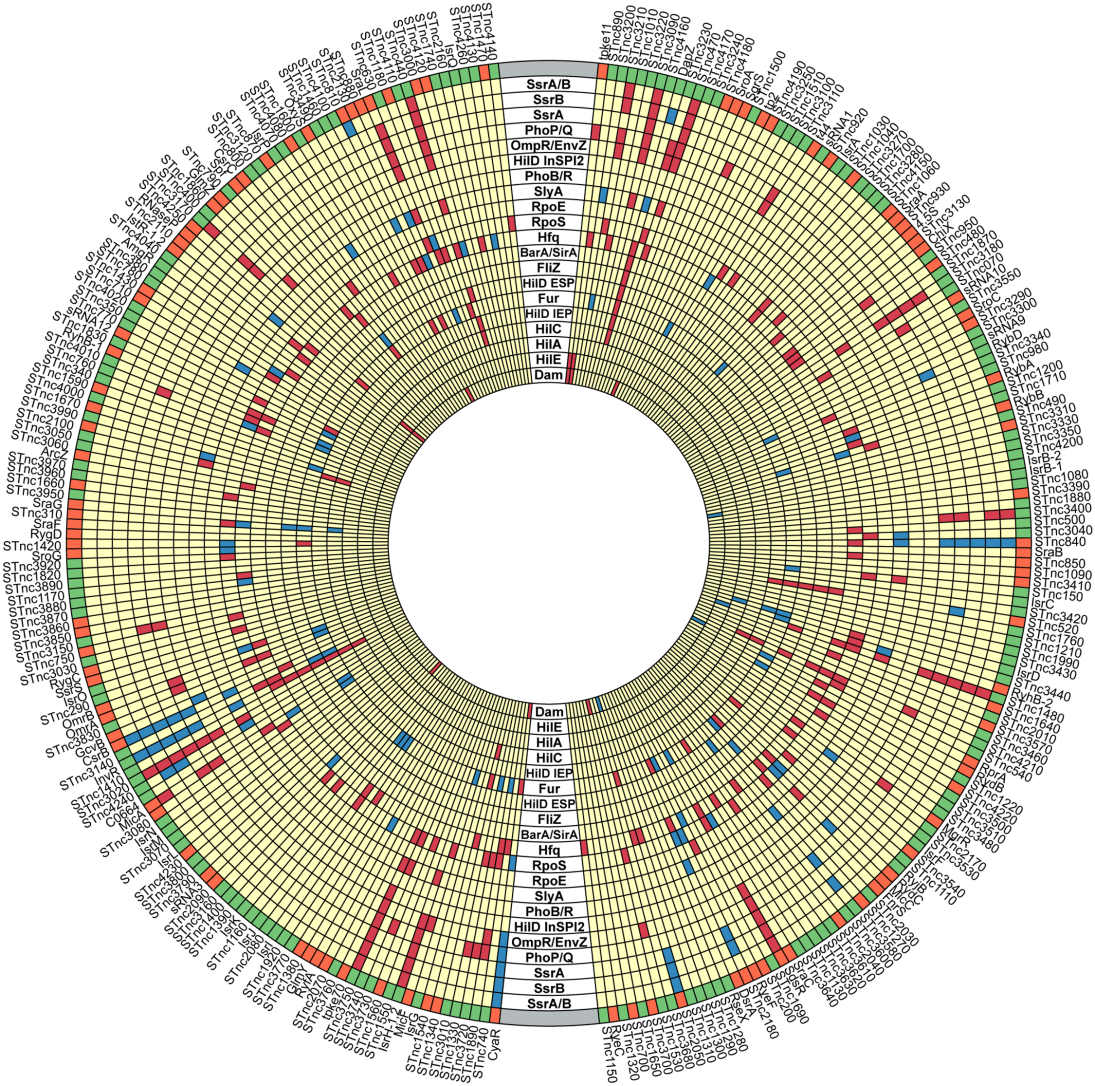
Pattern of differential expression of *S*. Typhimurium sRNAs in the panel of regulatory mutants. Concentric circle diagram representing all 280 *S*. Typhimurium sRNAs, which are labelled outside the circle according to their relative chromosomal position. The putative regulatory inputs reflect the pattern of sRNA gene expression in the panel of regulatory mutants. Each circular track represents a single regulatory system. Red boxes indicate putative activation of sRNA expression (>3-fold decrease in expression in mutant compared to wild-type); Blue boxes indicate putative repression of sRNA expression (>3-fold increase in expression in mutant compared to wild-type); yellow indicates no change in expression in mutant compared to wild-type. The outermost ring indicates the conservation status of each sRNA, with *Salmonella*-specific sRNAs shown in green. The sRNAs that are conserved in other enteric species are orange, based on our previous analysis [14]. Diagram generated with Circos (http://www.circos.ca).

The Transcripts Per Million (TPM) approach was used to generate expression values from the RNA-seq data, and to define whether individual genes were expressed (threshold = TPM 10). Approximately 75% of the 280 sRNAs of wild-type *S*. Typhimurium strain 4/74 were expressed in all 5 conditions used in this study, while a further 20% of sRNAs were expressed in at least one environmental condition (Fig 4A). In the panel of transcription factor mutants, 44% of *S*. Typhimurium sRNA genes (124) were differentially-expressed by at least 3-fold. Almost 50% of the differentially-expressed sRNAs received only one regulatory input (direct or indirect) from the panel of TFs and approximately 11% of differentially-expressed sRNAs received 5 or more regulatory inputs from the panel of TFs (Fig 4B). The sRNA genes in each of the categories shown in Fig 4B are detailed in S6 Table. Fig 5 shows the shared sRNA targets between each regulatory system. We identified many sRNAs that are controlled by both the SPI1 and SPI2 regulatory systems, reflecting the hierarchical regulatory structures that control expression of the two main pathogenicity islands.

The majority (>60%) of differentially-expressed sRNAs were transcribed from intergenic regions, while over 20% of differentially-expressed sRNAs were located in the 3’UTR of a coding gene, a chromosomal location that constitutes a large reservoir for small non-coding RNAs [51]. Forty-eight percent of the 3’UTR-derived sRNAs are transcribed from their own promoter, while the remaining 52% are likely to be processed from the mRNA of the upstream ORF [9]. We speculate that processed sRNAs are often functionally-related to the upstream co-transcribed ORFs, while expression of the sRNAs that are transcribed from their own promoter may be controlled by alternative transcription factors and these sRNAs have a distinct function. Expression of the majority (57%) of ORFs upstream of putative processed sRNAs follow the same regulatory pattern as their 3’-derived sRNAs in the panel of regulatory mutants (>3-fold change; S6 Table), while only one upstream ORF displays the same regulatory pattern as its non-processed downstream 3’UTR-derived sRNA (S6 Table). Over 60% of differentially-expressed 3’-encoded sRNAs were Hfq-enriched [51], suggesting that many of the RNAs in this category function as canonical *trans*-acting sRNAs and require the RNA chaperone Hfq. Conversely, only 20% of antisense-encoded sRNAs were enriched for Hfq binding, highlighting the fact that many of this class of sRNAs are Hfq-independent and may act in *cis* to their target genes (S3 Fig and S6 Table).

**Fig 6:**
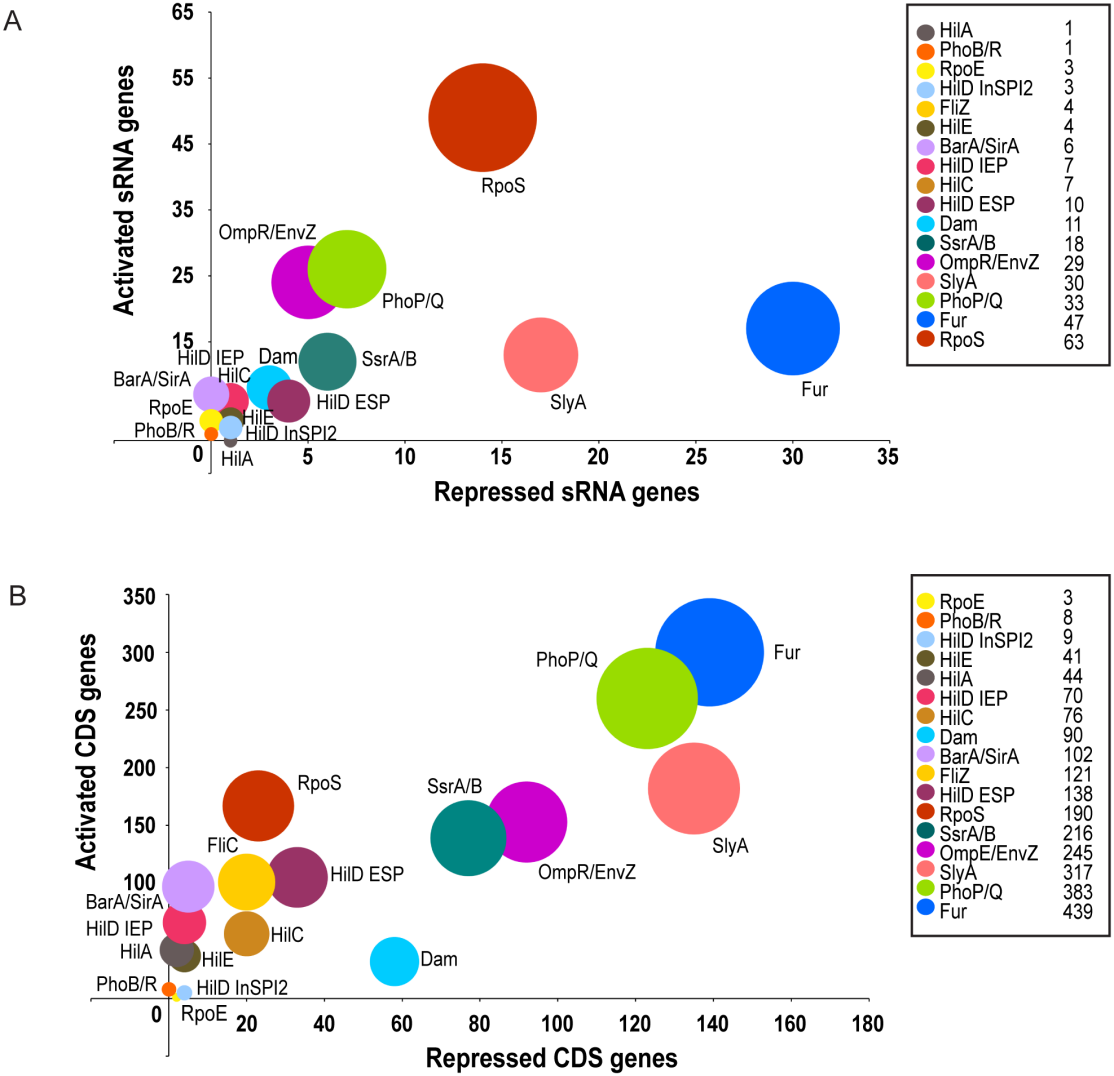
sRNA and CDS expression in the panel of regulatory mutants. Bubble diagram showing the numbers of differentially-expressed sRNA genes (A) and the numbers of differentially-expressed CDS (B) in the panel of regulatory mutants. The bubble size is proportional to the number of differentially-expressed genes in the isogenic mutant that lacks each regulatory system. The x-axis indicates the number of genes which show increased expression (repressed) in the mutant strain, the y-axis indicates the number of genes which show decreased expression (activated) in the mutant strain. The numbers of differentially-expressed genes are indicated in the key to the right of each graph.

The size of the sRNA-based regulons ranges from 63 differentially-expressed sRNAs (RpoS) to one differentially-expressed sRNA (HilA and PhoB/R) (Fig 6A and S4A Fig). The SPI1-encoded regulators HilC and HilD function as part of a feed-forward regulatory loop [4], and considerable overlap was observed between the sRNA genes that were controlled by these TFs (Fig 5). The HilE CDS and sRNA regulons were distinct from the HilA, HilC and HilD regulons at IEP, and show a novel positive regulatory role for HilE in the control of metabolic gene expression (S3 Table), in addition to the reported role as a negative regulator of SPI1 genes [52]. The sRNA genes which were differentially-expressed in the absence of the BarA/SirA regulatory system were a subset of the HilC and HilD-regulated genes, reflecting the indirect activation of SPI1 by BarA/SirA, via the sRNAs CsrB and CsrC (Fig 5) [18]. More sRNA genes were up-regulated than down-regulated in the mutant strain that lacks Fur, consistent with the primary function of Fur as a transcriptional repressor [53]. RyhB-1 and RyhB-2, the *S*. Typhimurium homologues of the *E*. *coli* Fur-repressed sRNA RyhB, were up-regulated in the absence of Fur.

We investigated whether promoters of sRNAs which were up-regulated in the absence of Fur contained “Fur-boxes” or recognition motifs for Fur binding, consistent with direct repression of expression by Fur. The promoters of five up-regulated sRNA genes contained high-scoring putative Fur boxes (S5 Fig). The highest scoring Fur box was present in the promoter of the Fur-dependent RyhB-1 gene [54]. Other high-scoring Fur boxes were located in the promoters of sRNA genes located adjacent to iron-associated or Fur-regulated operons. STnc4000 contains a putative Fur binding site overlapping the -10 site of the promoter region. This sRNA is encoded in an intergenic region and is transcribed divergently from the *bfd* gene, which encodes a Fur-regulated bacterioferritin-associated ferredoxin protein. The second highest scoring putative Fur binding site was obtained for a sequence overlapping the -10 site of the STnc3250 promoter. STnc3250 is intergenic and transcribed divergently from *fhuA*, which is the first gene in a Fur-dependent operon that encodes ferrichrome-iron associated proteins. The proximity of STnc4000 and STnc3250 to genes involved in iron homeostasis raises the possibility that Fur regulation of STnc4000 and STnc3250 may be involved in the control of iron metabolism. The Fur-repressed divergently-oriented promoters of STnc3250 and STnc4000 may represent new examples of the bidirectional promoters that belong to the Fur regulon, such as, *fepA-fes*, *fepD-ybdA* and *fepB-entCEBA* [55].

We found that SPI2-associated regulators OmpR/EnvZ, PhoPQ, SsrAB and SlyA control more sRNAs than the SPI1-associated regulators (Figs 5 and 6A and S4A Fig). Many of the sRNA genes which are differentially-expressed in the SPI2-associated regulatory mutants are likely to be indirectly regulated, reflecting the complexities of the SPI2 regulatory hierarchy [56]. The sRNAs which are differentially-expressed in the absence of SsrA/B are a subset of those that are differentially-expressed in the absence of the PhoP/Q and OmpR/EnvZ two-component systems (TCS) (Fig 5), reflecting the roles that PhoP and OmpR play in the control of virulence gene expression as well as the regulation of components of the ancestral genome [57, 58]. STnc1860 was the only differentially-expressed sRNA in the Δ*phoB/R* mutant (>4-fold decrease in expression). STnc1860 is located downstream of the *phoU* gene which encodes a transcriptional regulator of the PstSCAB-PhoU high affinity phosphate transport system operon and is a target of the PhoB/R TCS [59]. STnc1860 is co-transcribed with this operon from the *pstS* start site [9] and could be involved in the control of phosphate assimilation.

Overall, each regulatory system modulated the expression of a similar proportion of sRNAs and CDS (Fig 6B and S4B Fig). The general effect of the panel of regulatory mutations is negative on expression of CDS and sRNA genes. More differentially-expressed CDS genes were controlled by the SPI2-associated regulatory systems than the SPI1-associated regulators. The key difference between the sRNA-based and CDS-based regulatory networks was seen in the context of RpoS-dependent transcriptional control. RpoS modulated the expression of more sRNA genes than other regulatory proteins (S4B Fig), whereas the CDS-based network of RpoS was among the smaller regulons, containing only 190 putative RpoS-dependent coding genes. We propose that RpoS is a hub for sRNA-mediated gene regulation in *S*. Typhimurium.

RpoS-regulated genes play a role in the general stress response at LSP [60]. The prominent role played by RpoS in regulation of sRNA expression at LSP may reflect the pleiotropic functions that sRNAs play in mediating cellular responses to stress [16], or the lack of expression of many CDS under LSP conditions. The large number of sRNAs which were differentially-expressed in the absence of RpoS highlights the importance of RpoS as a hub for post-transcriptional regulation [61–63]. Of the 63 sRNAs that are differentially-expressed in the absence of RpoS, 36 are *Salmonella*-specific and 27 are conserved in other species [14] (Fig 5). ChIP analysis has shown that three sRNAs, OmrA, SibC (RygC) and RyeB (SdsR) belong to the core RpoS regulon of *E*. *coli* [64]. These three conserved sRNAs were also differentially-expressed in the absence of RpoS in our study (S3 Table). Furthermore, Peano *et al*. identified an RpoS binding site in the intergenic region between the divergently transcribed *tisB* gene and the conserved sRNA IstR-1_2. The expression of IstR-1_2 is reduced in the absence of RpoS in our study, suggesting that RpoS activates IstR-1_2 expression in both species. We anticipate that, in future, the joint interrogation of our transcriptomic data with other global ChIP-based studies will elucidate entire regulons.

We aligned the promoter sequences of the 49 sRNAs that were down-regulated (>3-fold) in the absence of RpoS to determine if the promoters of the *Salmonella*-specific sRNAs contain hallmarks of RpoS-dependent promoters and are, therefore, likely to be genuine members of the RpoS regulon. A C nucleotide at position -13 relative to the transcriptional start site is highly conserved among RpoS-dependent promoters, and does not favour binding by RpoD [65]. Twenty-one of the 49 RpoS-dependent promoters contained a C at position -13, including the promoters responsible for the expression of OmrA, SdsR and IstR-1_2, (S6 Fig). Placing of a G or T at position -14 and degeneracy of the sequence and position of the -35 hexamer are also typical of RpoS-dependent promoters [66] and are common features of the RpoS-dependent sRNA promoters (S6 Fig).

sRNA promoters are not qualitatively different from the promoters of CDS [67] and, with the exception of the RpoS regulon, the striking similarities of regulatory input on sRNA promoters with the regulatory input on CDS promoters demonstrate that the transcriptional regulation of sRNAs is mediated by established cellular regulatory networks. Our panel of *S*. Typhimurium regulatory systems did not reveal a single dedicated TF for transcriptional control of all sRNAs, suggesting that sRNAs are likely to have been integrated into existing networks as required.

### Confirmation of key regulatory inputs for five sRNAs

**Fig 7:**
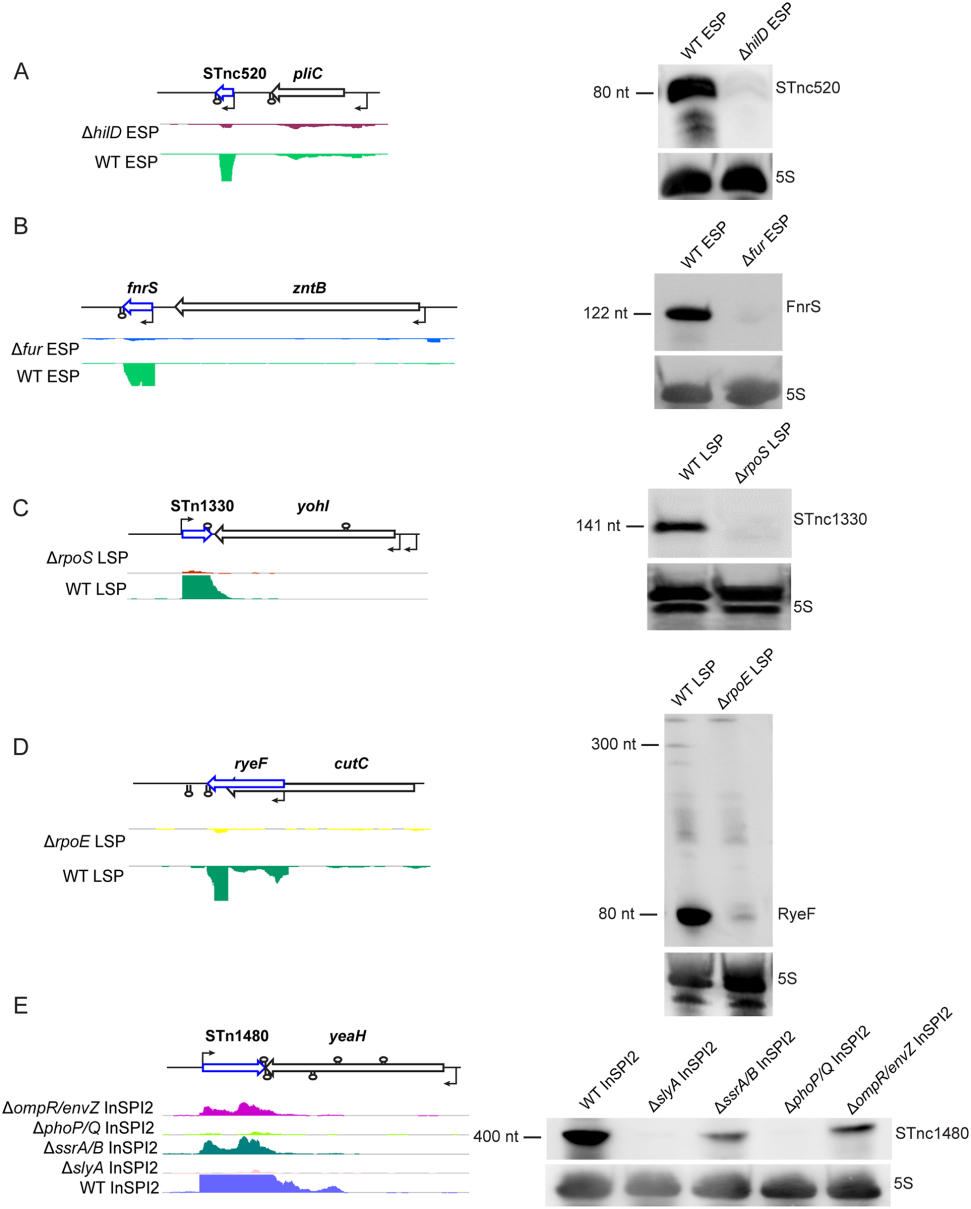
Confirmation of novel regulatory interactions that control five sRNAs. (A) STnc520, (B) FnrS, (C) STnc1330, (D) RyeF, (E) STnc1480. Each panel shows the visualisation of sequencing reads obtained from RNA-seq experiments using the IGB browser beside northern blots that validate the differential expression data using RNA extracted from biological replicates of wild-type and mutant strains grown under the same *in vitro* environmental condition, as indicated. 5S RNA was probed as a loading control.

To confirm the regulatory findings for sRNAs of interest, we used northern blots to confirm differential sRNA gene expression between wild-type and regulatory mutants (Fig 7). Expression of the sRNA STnc520 decreased 17-fold in the absence of the primary SPI1 regulator, HilD, at ESP (Fig 7A). In *S*. Typhimurium strain 14028, expression of STnc520 was directly activated by the SPI1-encoded transcription factor, SprB (Joseph T. Wade, pers. comm.). Direct regulation of STnc520 by SprB was also confirmed in *S*. Typhimurium 4/74 in this study (S7A and S7B Fig).

Expression of the FNR-dependent sRNA FnrS decreased approximately 32-fold in the absence of Fur at ESP (Fig 7B), consistent with the reported overlap between the Fur and FNR regulons [45]. The Fur-mediated up-regulation of FnrS is unlikely to be a direct effect, however, as Fur does not directly activate transcription [53]. The wild-type strain accumulates the sRNA STnc1330 at LSP and, in keeping with previous findings [63], STnc1330 expression is reduced approximately 61-fold in the absence of RpoS (Fig 7C). RyeF expression was reduced 25-fold in the absence of *S*. Typhimurium RpoE (Fig 7D) and we note that MicL, the *E*. *coli* orthologue of the RyeF sRNA, belongs to the RpoE regulon in *E*. *coli* [68].

The *Salmonella*-specific sRNA, STnc1480, was most highly expressed under environmental conditions that mimic the host intracellular environment and within murine macrophages [9, 14]. STnc1480 has multiple regulatory inputs from SPI2-associated regulatory systems, with the largest reduction in STnc1480 expression (approximately 58-fold) being seen in the absence of both the PhoP/Q and SlyA regulatory systems (Fig 7E).

### Transcriptional regulation of the STnc1480 sRNA

**Fig 8:**
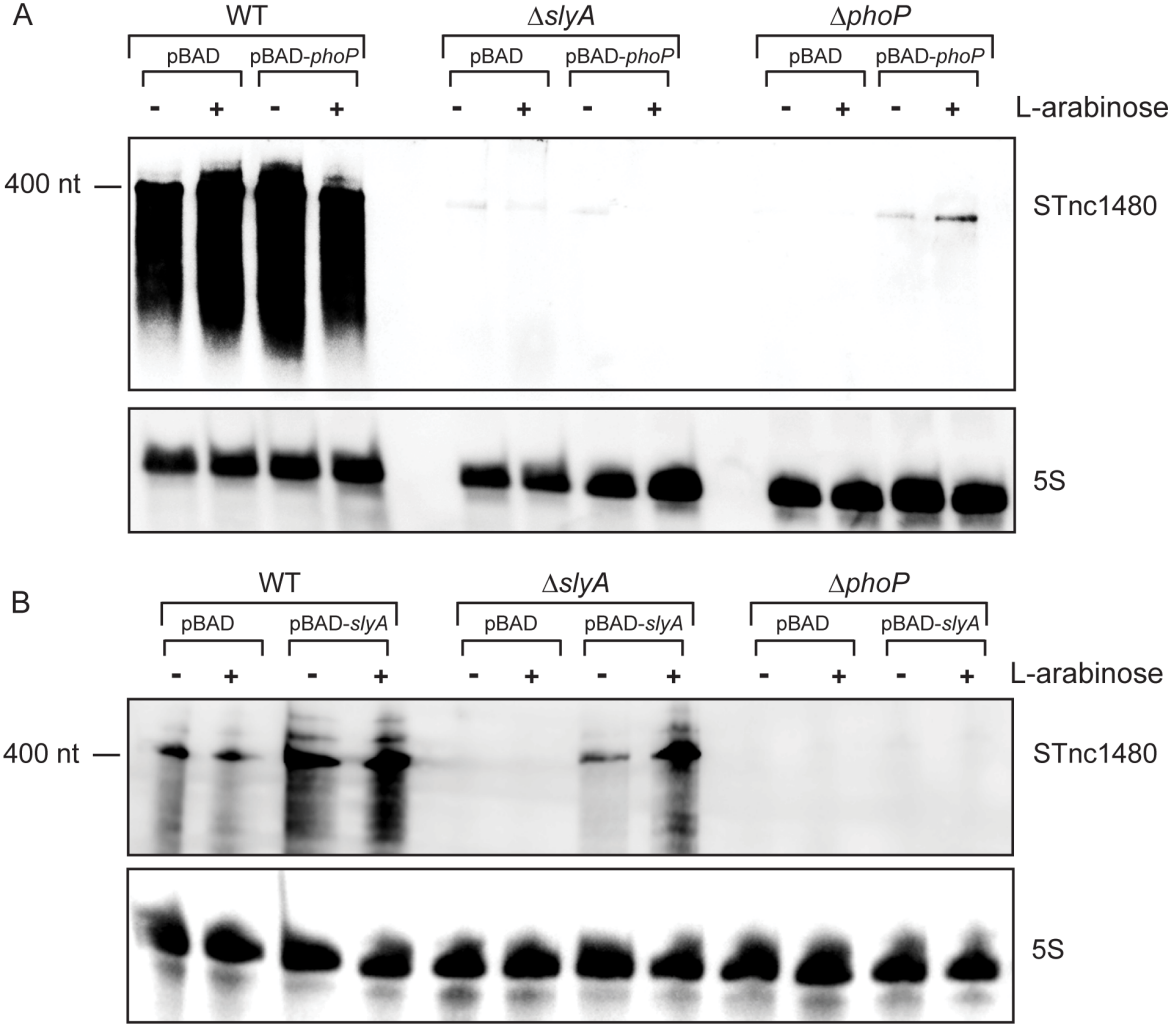
Regulation of STnc1480 expression. (A) Northern blot showing that STnc1480 is expressed in wild-type cells and is SlyA-dependent, even when PhoP expression is induced from the P_BAD_ promoter. STnc1480 expression is restored when PhoP is ectopically expressed in a Δ*phoP* background and addition of L-arabinose to a Δ*phoP* mutant carrying the empty pBAD vector does not affect STnc1480 expression. (B) Northern blot showing that STnc1480 is expressed in wild-type cells and accumulates to a higher level when SlyA is ectopically expressed from the P_BAD_ promoter. STnc1480 expression was rescued when SlyA was exogenously supplied in a Δ*slyA* mutant and addition of L-arabinose to a Δ*slyA* mutant carrying the empty pBAD vector does not affect STnc1480 expression. STnc1480 expression was not rescued by the ectopic expression of SlyA in the absence of PhoP. -/+ indicates the respective absence or presence of the inducer L-arabinose. 5S RNA was probed as a loading control. Each northern blot is representative of at least 3 independent experiments.

The expression and regulatory profile of STnc1480 suggests that this sRNA may be important during the intracellular lifestyle of *Salmonella*, leading us to investigate the expression pattern of this sRNA in greater detail. The PhoP and SlyA-dependent expression of STnc1480 was confirmed by ectopic expression of PhoP or SlyA from the arabinose-inducible P_BAD_ promoter, which specifically restored STnc1480 expression in Δ*phoP* and Δ*slyA* mutant backgrounds respectively; however ectopic expression of either protein was unable to restore STnc1480 expression in a mutant strain which did not express the other regulator (Fig 8A and 8B). These data argue that both SlyA and PhoP are required for optimal expression of STnc1480. There is significant overlap between the SlyA and PhoP regulons and a transcriptional requirement for both SlyA and PhoP has previously been reported for horizontally-acquired genes that are subject to silencing by the nucleoid-associated protein H-NS [44, 69, 70]. We, therefore, determined whether STnc1480 transcription was subject to H-NS-mediated silencing and subsequent counter-silencing by SlyA and PhoP.

**Fig 9:**
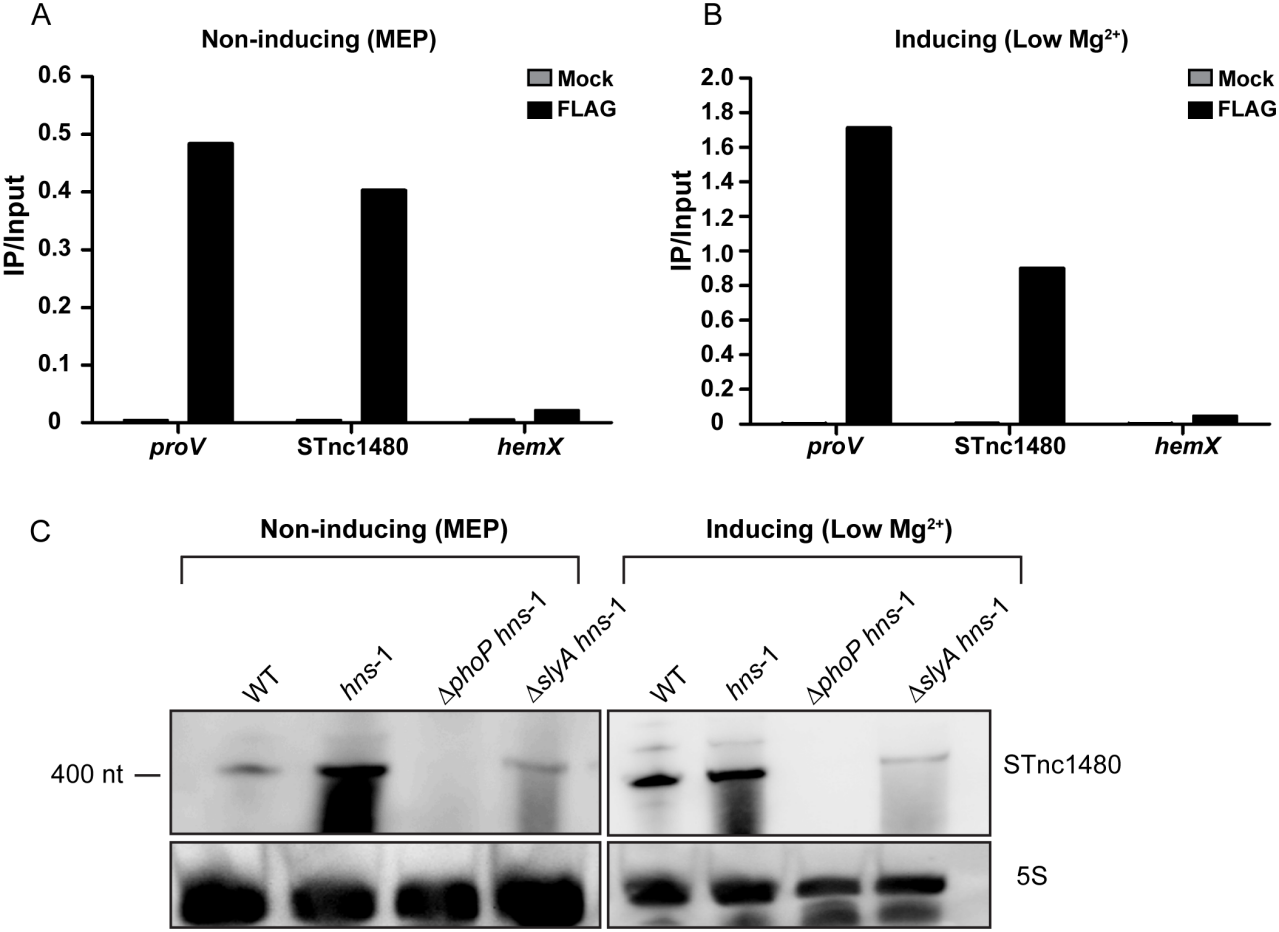
H-NS occupancy of the STnc1480 promoter. qPCR data of representative ChIP assay from two independent experiments demonstrating H-NS binding to the STnc1480 promoter under (A) non-inducing (MEP) and (B) inducing (LowMg^2+^) environmental conditions. Experimental (FLAG) and mock DNA from the STnc1480 promoter and the positive (*proV*) and negative (*hemX*) control regions was normalised to the starting amount of DNA, which was extracted prior to immunoprecipitation (IP/Input). (C) Northern blot showing expression of STnc1480 under non-inducing and inducing conditions in wild-type and in the absence of a functional H-NS protein in combination with Δ*slyA* and Δ*phoP* mutations. 5S RNA was probed as a loading control.

Chromatin immunoprecipitation followed by qPCR was used to investigate H-NS occupancy of the STnc1480 promoter under non-inducing conditions (MEP) and inducing conditions (Low Mg^2+^). The *proV* promoter and the *hemX* gene were used as positive and negative control regions, respectively, as H-NS binds to the *proV* promoter and does not bind to the *hemX* gene [71]. Fig 9A and 9B are representative of two independent biological replicates and show that H-NS associated with the STnc1480 promoter, with greater enrichment observed for the experimental IP sample (FLAG), compared to the mock IP sample under both inducing and non-inducing conditions. These data indicate that H-NS bound directly to the STnc1480 promoter under non-inducing conditions and that H-NS was not displaced from the STnc1480 promoter under conditions when the sRNA was highly expressed.

To further understand the relationship between STnc1480 and the TFs PhoP and SlyA, and to characterise the roles of PhoP and SlyA in counter-silencing, we investigated the expression of STnc1480 in strains lacking a functional H-NS protein in either Δ*phoP* or Δ*slyA* mutant backgrounds. Fig 9C confirms that STnc1480 was not highly expressed during logarithmic growth in rich medium in a wild-type background, and that STnc1480 expression was de-repressed in the absence of a functional H-NS protein under this growth condition. SlyA was no longer fully required for STnc1480 transcription under non-inducing or inducing conditions, arguing that the key role of SlyA in STnc1480 expression is to counteract the repressive effects of H-NS, rather than to activate transcription; SlyA plays a similar function at the *pagC* promoter [70]. Counter-silencing of H-NS is likely to be achieved through SlyA-mediated restructuring of the STnc1480 promoter architecture, rather than displacement of H-NS. We speculate that PhoP plays the role of a classical transcriptional activator at the STnc1480 promoter, rather than counter-silencer, as the presence of PhoP is required for transcription even in the absence of a functional H-NS protein.

### Identification of sRNAs with SPI1-like and SPI2-like expression profiles

The “guilt by association” hypothesis posits that groups of genes which perform similar functions are co-expressed and/or co-regulated, allowing transcriptomic data to be used to identify genes which may share related functions [72]. Several approaches are available to reveal novel interactions between TF and co-expressed genes [73], and correlative patterns are becoming widely used for the inference of causal influence and to define transcriptional networks [74]. We previously identified transcriptional signatures for SPI1- and SPI2-related genes, based on the expression profiles of the archetypical SPI1 gene, *prgH*, and the archetypical SPI2 gene, *ssaG* [9]. To identify sRNA genes which may play important roles in *S*. Typhimurium virulence, we searched for sRNAs with expression profiles that closely correlate to the expression of SPI1 and SPI2 genes.

**Fig 10:**
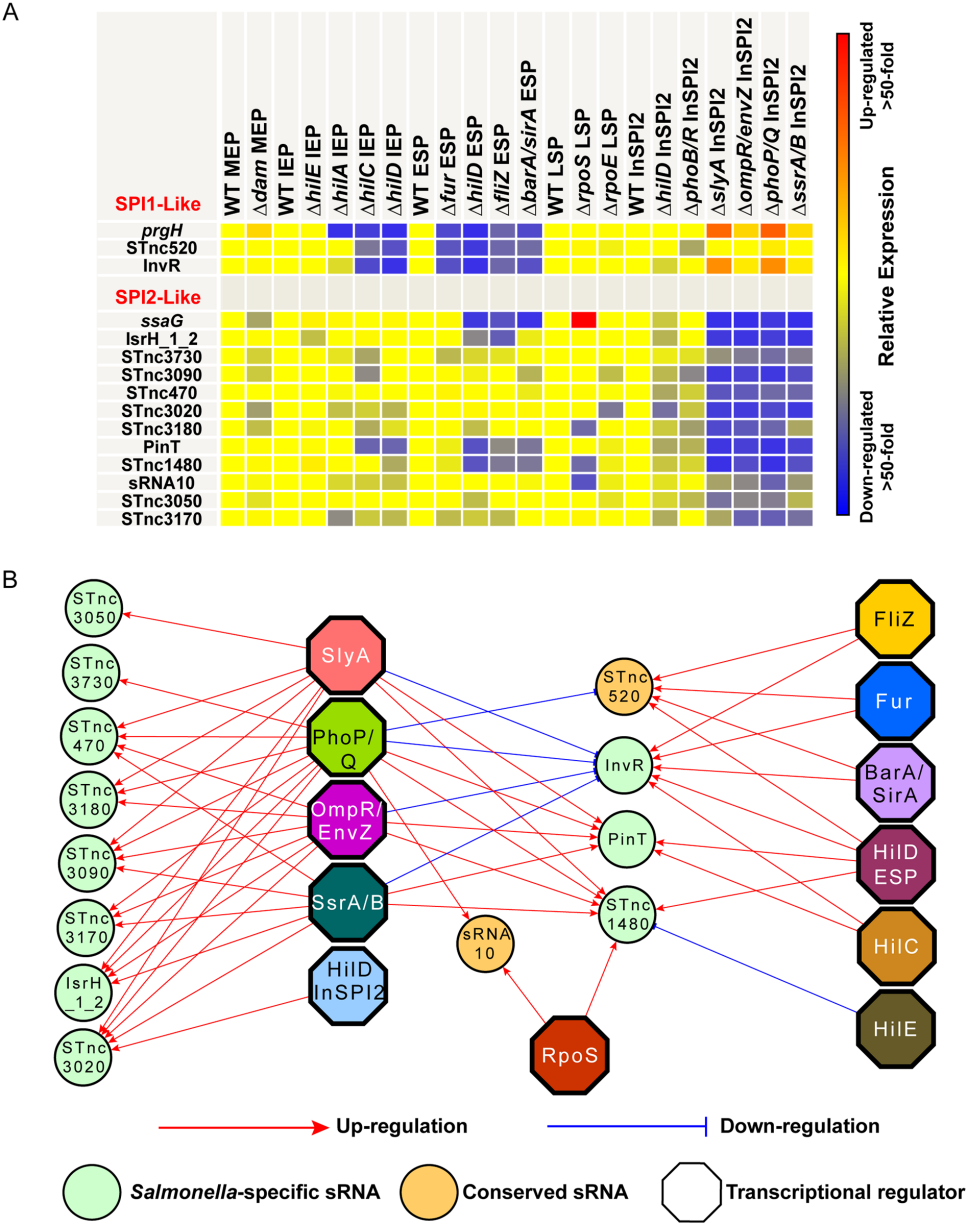
Putative regulatory inputs for thirteen SPI1-like and SPI2-like sRNAs. (A) Heatmap showing relative expression of two SPI1-like sRNAs in comparison to the archetypical SPI1 gene *prgH* and 11 SPI2-like sRNAs in comparison to the archetypical SPI2 gene *ssaG* in a panel of 15 regulatory mutants, compared to the wild-type strain grown under the same environmental condition. SPI1-like sRNAs were defined as sRNAs which show a similar pattern of expression (Pearson correlation coefficient >0.7) under 22 *in vitro* environmental conditions, within murine macrophages and in a panel of 18 regulatory mutants to the archetypical SPI1 *prgH* gene. SPI2-like sRNAs were defined under exactly the same conditions using the archetypical SPI2 gene *ssaG*. Yellow indicates no change in expression; red indicates an increase in expression; and blue indicates a decrease in expression in the mutant strain, compared to wild-type. (B) Regulatory network representing a hypothetical model of the transcriptional regulation of the SPI1-like and SPI2-like sRNAs, based on differential expression in the absence of the indicated regulatory protein. *Salmonella*-specific sRNAs are green circles; *Enterobacteriaceae*-conserved sRNAs are orange circles; transcription factors are multi-coloured octagons; up-regulation (>3-fold decrease in expression in mutant/wild-type) shown with a red arrow; down-regulation (>3-fold increase in expression in mutant/wild-type) shown with a blue T-bar. Regulatory network was generated using www.cytoscape.org.

Our global transcriptomic analyses identified 2 sRNA genes that showed a SPI1-like pattern of expression across 22 environmental conditions [9], within murine macrophages [14] and in our panel of regulatory mutants (Pearson correlation coefficient > 0.7 [*prgH*]) (Table 4 and Fig 10A). InvR (located within SPI1) is transcriptionally activated by the primary SPI1 transcription factor, HilD [75], confirming the value of correlative analysis for identifying genuine regulatory interactions. As previously discussed, the second SPI1-like sRNA, STnc520, is directly regulated by SPI1-encoded SprB.

**Table 4:** sRNAs which show co-regulation with SPI1 or SPI2.

**SPI1-like sRNA**	**Upstream Gene**	**Downstream Gene**	**Genomic context**	**Pearson correlation coefficient with *prgH***^a^	**Significant TraDIS attenuation**
STnc520	*pliC*	*pagC*	*> < <*	0.84	Yes
InvR	*invH*	STM2901	> > >	0.75	Yes
**SPI2-like sRNA**	**Upstream Gene**	**Downstream Gene**	**Genomic context**	**Pearson correlation coefficient with *ssaG***^b^	**Significant TraDIS attenuation**
IsrH_1_2	*sseL*	*glpC*	> < >	0.98	Yes
STnc3730	*yfcC*	*pta*	> < >	0.89	- ^c^
STnc3090	*oadG*	STM0057	< > <	0.87	-
STnc470 (InvS)	STM0082	STM0081	> < <	0.85	-
STnc3020	*prgJ*	*prgH*	as to *prgI*	0.84	Yes
STnc3180	*ybdO*	*dsbG*	< > <	0.83	-
PinT	STM4310	STM4312	> > <	0.82	-
STnc1480	*yeaJ*	*yeaH*	< > <	0.77	Yes
STnc3050	*oadG*	SL3326	< < <	0.75	Yes
sRNA10	*Rnk*	*Rna*	< < <	0.75	-
STnc3170	*intA*	*thdF*	> < >	0.72	-

^a^ Pearson correlation coefficient with *prgH* across 22 environmental conditions, within murine macrophages and in panel of 18 regulatory mutants

^b^ Pearson correlation coefficient with *ssaG* across 22 environmental conditions, within murine macrophages and in panel of 18 regulatory mutants

^c^-indicates either that there was either no transposon insertion within this gene, or that there was a transposon insertion that was not associated with significant attenuation.

Eleven sRNA transcripts showed a SPI2-like expression pattern (Pearson correlation coefficient > 0.7 [*ssaG*]) (Table 4 and Fig 10A). None of the SPI2-like sRNA genes are located in the SPI2 pathogenicity island. In fact, the SPI2-like sRNA STnc3020 is encoded within the SPI1 island, antisense to *prgI*, and shows a modest negative correlation with expression of the archetypical SPI1 gene, *prgH* (Pearson correlation coefficient: -0.21). A high scoring SsrB binding motif was identified adjacent to the putative -35 site of the STnc3020 promoter (S8 Fig). This observation raises the intriguing possibility that STnc3020 has been co-opted by SsrB to integrate regulatory and environmental cues and mediate cross-talk between SPI1 and SPI2.

The STnc1480 sRNA shows a SPI2-like expression profile [9]. The previously-discussed PhoP- and SlyA-dependence of this sRNA suggests that STnc1480 could play a role in the expression of SPI2-associated regulatory systems. Furthermore, a second SPI2-like sRNA, PinT (STnc440) is PhoP-dependent, and was one of the most highly up-regulated sRNA transcripts upon internalisation of *Salmonella* within macrophages [14] and various other host cell types [76]. PinT represses both SPI1- and SPI2-associated virulence genes, and is a post-transcriptional timer of *Salmonella* gene expression during infection [76].

We speculated that some uncharacterised sRNAs that show SPI1 or SPI2-like expression patterns could have important functions in *S*. Typhimurium virulence and we investigated potential virulence phenotypes in the context of TraDIS datasets [41]. The two SPI1-like and four SPI2-like sRNAs were required for optimal fitness during infection of the chicken, pig or calf models (summarised in Table 4).

We present a hypothetical model of a regulatory network which highlights the key regulatory inputs (>3-fold change in expression) of the 13 SPI1-like and SPI2-like sRNAs (Fig 10B), showing direct or indirect regulation of the SPI1-like and SPI2-like sRNAs mainly by SPI-associated regulatory proteins. The two SPI1-like sRNAs, InvR and STnc520, receive negative regulatory inputs from the SPI2-associated regulatory proteins in addition to the positive regulatory inputs from the SPI1-associated regulators, consistent with cross-talk between the expression of the SPI1 and SPI2 systems. The 11 SPI2-like sRNAs are highly interconnected by their regulatory inputs reflecting the complexity of the SPI2 regulatory hierarchy, although a number of the regulatory interactions are likely to be indirect. This hypothetical regulatory network provides the foundation for future experimentation to identify the direct mechanism of transcriptional regulation of the SPI1-like and SPI2-like sRNAs, which present interesting candidates for further analysis.

### Conservation of SPI1-like and SPI2-like sRNAs

Our laboratory recently published a phylogenetic analysis of the 280 *Salmonella* sRNAs across 29 enterobacterial genomes [14]. One hundred and seventy-six “*Salmonella*-specific” sRNAs were identified, that showed >90% sequence identity across the *Salmonella* genus and <70% sequence identity with other members of the *Enterobacteriaceae*. Eleven of the 13 SPI1-like and SPI2-like sRNAs were *Salmonella*-specific. This high proportion of *Salmonella*-specific sRNAs is consistent with a role for the SPI1-like and SPI2-like sRNAs in the evolution of *S*. Typhimurium as a pathogen (S5 Table). Despite the fact that none of the 11 SPI2-like sRNAs are encoded within the SPI2 island, six SPI2-like sRNAs are *S*. *enterica*-specific and are not conserved in *Salmonella bongori*. The limited conservation of these sRNAs outwith the *S*. *enterica* species suggests that these elements were either acquired with or after their SPI2-encoded regulators, and were co-opted to perform regulatory functions within the intracellular environment after the evolutionary divergence of *S*. *enterica* and *S*. *bongori*. We performed a similar phylogenetic analysis of the 15 TFs, response regulators and sigma factors used in this study and confirmed that HilD, HilA, HilC, HilE and SsrB are *Salmonella*-specific regulators, while the remaining 10 regulators are conserved outwith the *Salmonella* genus (S5 Table). We found that the SPI1-like and SPI2-like sRNAs are controlled by a mixture of regulatory inputs from both *Salmonella*-specific and *Enterobacteriaceae*-conserved regulatory systems.

### The Hfq regulon

The RNA chaperone Hfq facilitates binding between sRNA and target mRNA molecules and contributes to sRNA-mediated post-transcriptional regulation by various mechanisms. Hfq also controls sRNA stability prior to target recognition, either through protection from ribonucleases or through promotion of sRNA decay [77]. The role of Hfq in modulating the global expression of *Salmonella* sRNAs has not yet been reported as our previous analysis of the *Salmonella* Hfq regulon relied upon DNA microarrays [78]. Previous co-immunoprecipitation analysis, combined with RNA-seq, demonstrated that approximately half of *S*. Typhimurium sRNAs were associated with Hfq in a number of environmental conditions [51]. This led us to investigate how the absence of the Hfq protein affects the steady state levels of the Hfq-bound and non-bound sRNAs. Clearly, interactions between Hfq and other proteins or with many mRNAs have pleiotropic effects on gene expression, and these effects cannot always be directly attributed to Hfq [79]. Therefore, we combined the available Hfq co-immunoprecipitation data [51] with our transcriptomic data to identify the Hfq-dependent sRNAs that were physically associated with Hfq, and represent candidate canonical *trans*-acting sRNAs (S6 Table).

**Fig 11:**
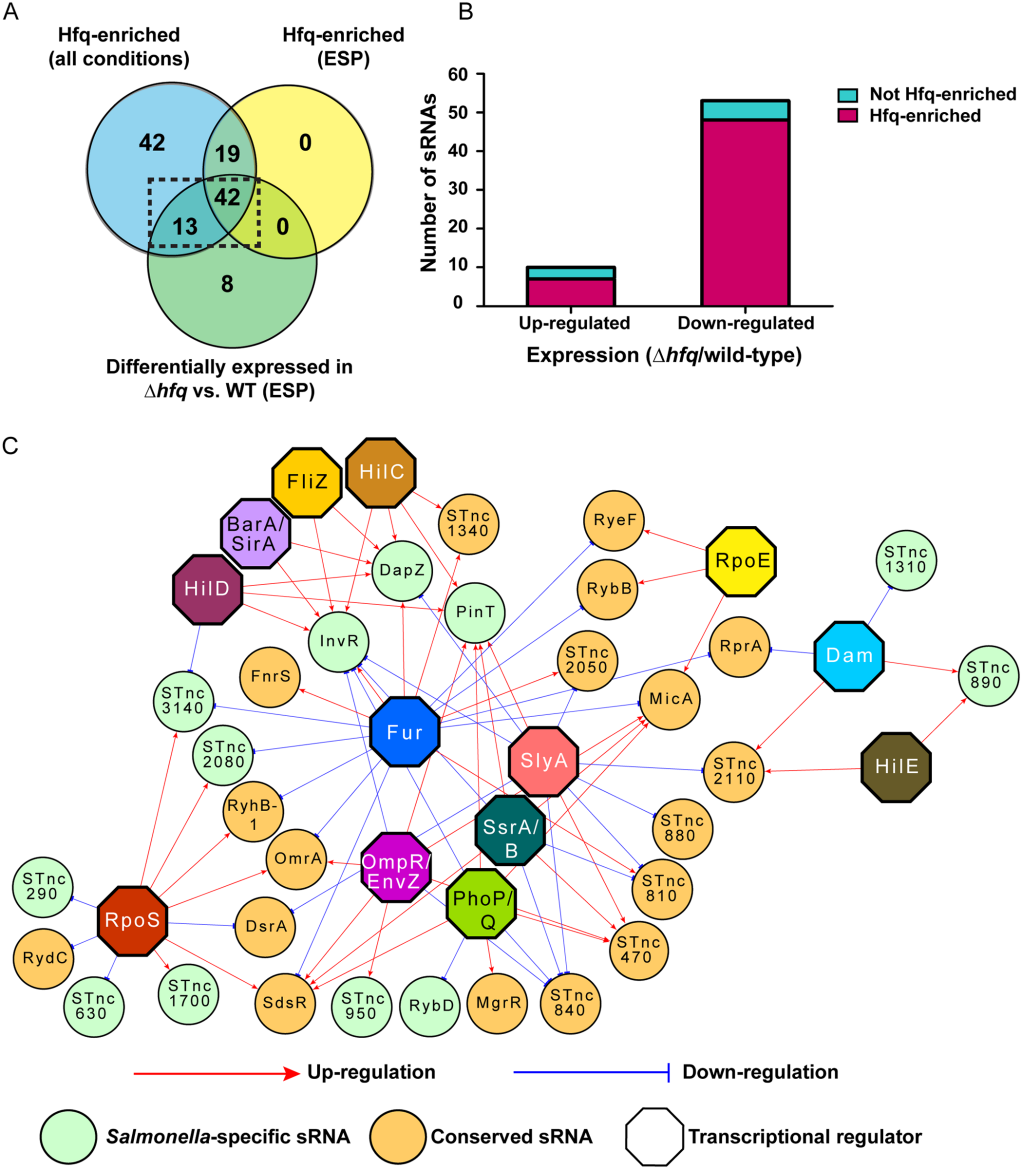
Analysis of the Hfq-dependent sRNA regulon. (A) Venn diagram comparing sRNA genes that were differentially-expressed in Δ*hfq* compared to wild-type, and sRNAs which were associated with Hfq under any condition, or specifically at ESP. Fifty-five sRNAs indicated within the dashed black box were included for analysis of differential expression in the panel of regulatory mutants for panel (C). (B) Bar chart demonstrating the number of sRNA genes that are up- or down-regulated in Δ*hfq* compared to wild-type in the context of association with Hfq. (C) Regulatory network representing a hypothetical model of the transcriptional regulation of 30 out of 55 Hfq-associated sRNAs, based on differential expression in the absence of the indicated regulatory protein. *Salmonella*-specific sRNAs are green circles; *Enterobacteriaceae*-conserved sRNAs are orange circles; transcription factors are multi-coloured octagons; up-regulation (>3-fold decrease in expression in mutant/wild-type) shown with a red arrow; down-regulation (>3-fold increase in expression in mutant/wild-type) shown with a blue T-bar. Association with Hfq was determined by Chao *et al* (2012) using Hfq co-immunoprecipitation [51].

Sixty-three of the 280 *S*. Typhimurium sRNAs were differentially-expressed in the absence of Hfq (3-fold or greater change in transcript level), and were designated as Hfq-regulated (S3 Table). The majority (87%; 55 sRNAs) of Hfq-regulated sRNA transcripts were enriched by co-immunoprecipitation with Hfq in at least one of the environmental conditions used by Chao *et al*, and 67% (42 sRNAs) were Hfq-enriched at ESP (Fig 11A).

Nineteen sRNAs were enriched for Hfq at ESP but did not show Hfq-dependent expression at ESP. Two of these sRNAs, AmgR and STnc2100, were not expressed at ESP or in the Δ*hfq* mutant and so were excluded from our analysis. The remaining 17 Hfq-enriched, non-differentially-expressed sRNAs may require Hfq binding for their activity and to aid binding to their target mRNAs but do not require Hfq for stability. A further 8 sRNAs were differentially-expressed in the Δ*hfq* mutant but not enriched for Hfq in any of the conditions used by Chao *et al*. The differential expression of a number of these sRNAs may have an indirect cause as the absence of Hfq has wide-ranging effects on sigma factors and TFs [78]. Therefore, the differential expression of some sRNAs in the Δ*hfq* mutant may reflect altered transcription rather than an altered rate of turnover. For example, approximately 16% of Hfq-regulated sRNAs were up-regulated in the absence of Hfq (Fig 11B), including the RpoE-dependent sRNAs RybB, MicA and RyeF [68, 80]. RpoE, and genes in the RpoE regulon, were more highly expressed in a Δ*hfq* mutant due to activation of the extra-cytoplasmic stress response [81], suggesting that increased levels of RpoE-dependent sRNAs in the Δ*hfq* mutant simply reflect an induction of RpoE. Approximately 30% of up-regulated sRNAs were not enriched for Hfq, compared to only 9% of down-regulated sRNAs which were not enriched for Hfq, reflecting an indirect regulatory effect on the up-regulated sRNA genes (Fig 11B).

We investigated how the 55 Hfq-regulated and Hfq-enriched sRNAs (highlighted using a dashed black box on Fig 11A) were expressed in the panel of regulatory mutants. The expression of thirty of the 55 Hfq-associated sRNAs was modulated by at least one of the regulatory systems under investigation. We generated a model of the transcriptional regulatory network for these thirty canonical *trans*-acting sRNAs, based on the sRNA expression patterns in the panel of regulatory mutants (Fig 11C). Approximately half of the Hfq-associated differentially regulated sRNAs were *Salmonella*-specific, while the other half are conserved in other members of the *Enterobacteriaceae* family (S5 Table). The potential for diversity of function of Hfq-dependent *trans*-acting sRNAs is reflected in the diverse range of transcriptional regulators of these sRNAs. The sRNAs with multiple regulatory inputs connect multiple hubs, and we speculate that these sRNAs play a physiological role in connecting regulons to integrate multiple regulatory signals and generate a co-ordinated genetic output *in vivo*. The sRNAs with fewer regulatory inputs may play more specific roles within their respective regulons.

This transcriptomic analysis of the Δ*hfq* mutant, coupled with the Hfq co-immunoprecipitation data [51], identified a core group of sRNAs that required Hfq for both their activity and stability. Many uncharacterised sRNAs belong to this core group, and represent interesting candidates for further investigation, which are likely to function as canonical *trans*-acting sRNAs under virulence-associated conditions.

### Community data resource

We previously developed an online tool, named SalComMac, which allows users to interrogate the expression profiles of *S*. Typhimurium genes in the wild-type strain grown under a suite of 22 infection-relevant environmental conditions [9] and within murine macrophages [14]. The RNA-seq sequence reads may be visualised in the context of the chromosome using the interactive online browser Jbrowse [9, 14]. We now provide the gene expression profiles of *S*. Typhimurium genes in the panel of regulatory mutants as a compendium database (http://tinyurl.com/SalComRegulon), and in the context of the chromosome (http://tinyurl.com/SalComRegulon-Jbrowse). The SalComRegulon tool provides absolute gene expression values in wild-type and mutant strains, and the relative expression of genes in mutant strains compared to the wild-type strain grown in the same environment. This transcriptomic dataset is intended to provide a valuable resource for the investigation of the expression profiles of genes of interest across a panel of regulatory mutants by the bacterial research community.

### Perspective

In the past, regulons have been defined in individual *Salmonella* strains, grown in various conditions and following different experimental criteria. Here, we have used a single *S*. Typhimurium strain, and just five growth conditions to perform a systematic and high-resolution analysis that offers the first opportunity for direct comparison between these infection-relevant regulatory systems. This analysis has allowed us to examine previously unseen regulatory interactions and uncover novel putative virulence genes. This database will provide the foundation for many hypothesis-driven future studies. We have expanded the regulons of key *S*. Typhimurium virulence-associated regulatory proteins under infection-relevant growth conditions and we present for the first time a detailed global view of the genes controlled by HilE, FliZ, RpoE and PhoB/R. The RNA-seq approach has given an unprecedented view of *S*. Typhimurium gene expression at single nucleotide resolution. By analysing the transcriptome of 18 mutants lacking important sigma factors, TFs, TCSs and an RNA chaperone we have identified new interactions between well-characterised regulatory systems, and the regulatory inputs that control the expression of the majority of uncharacterised *S*. Typhimurium sRNAs. As shown in S1 Fig, the regulatory systems under investigation in this study are key components of the complex transcriptional network responsible for the rapid adaptation required for successful infection of mammalian hosts.

The transcriptional control of sRNAs can generate regulatory loops which function via feed-forward and positive or negative feedback [82–84]. We predict that many of the 124 sRNAs that respond to the 18 regulatory systems will belong to new regulatory loops that link TFs to their target genes. The rapid kinetics of sRNA-mediated gene regulation [82, 83, 85–87] is likely to extend the flexibility and dynamic range of the regulatory systems of *Salmonella*. Future analysis of our transcriptomic data in conjunction with other datasets such as the environmentally-responsive and macrophage-regulated sRNA gene expression profiles [9, 14], global sRNA target identification and chromatin immunoprecipitation-derived TF binding sites will complete the picture of mixed regulatory interactions within the *Salmonella* cell. Here, the focus on the SPI1- and SPI2-associated regulons has identified 13 sRNAs that share the transcriptional signatures of *S*. Typhimurium virulence genes, including six sRNAs that are required for successful infection. We propose that these sRNAs control aspects of the pathogenesis of *S*. Typhimurium. The study takes us a step closer to the goal of elucidating the high-precision regulatory map of *S*. Typhimurium that allows this dangerous pathogen to cause hundreds of thousands of human deaths each year.

## Materials and Methods

### Bacterial strains and routine growth conditions

*Salmonella enterica* serovar Typhimurium strain 4/74 was used throughout this study [88]. All mutant strains and plasmids used in this study are listed in Table 1. Cells were routinely cultured in Lennox broth (10 g/L tryptone, 5 g/L sodium chloride, 5 g/L yeast extract). Unless otherwise stated, overnight cultures were sub-inoculated 1:1,000 in 25 mL of Lennox broth in a 250 mL Erlenmeyer flask with appropriate antibiotics. For growth in SPI2-inducing phosphate carbon nitrogen (PCN) medium [31], the inoculum was taken from overnight Lennox broth cultures, as previously described [9]. One mL of the overnight culture was harvested by centrifugation at 13,000 rpm at room temperature. The cells were washed 3 times in pre-warmed PCN medium and sub-inoculated 1:500 in 25 mL of minimal medium in a 250 mL flask with appropriate antibiotics. All cultures were incubated at 37°C and 220 rpm in an Innova 3100 water-bath shaker (New Brunswick Scientific).

### Growth curves

Relative growth rates of wild-type and mutant strains were determined in Lennox and PCN media using a Synergy H1 plate reader (Biotek). Overnight cultures were grown as described above. After overnight growth, strains grown in Lennox medium were diluted in fresh Lennox medium to a normalised OD_600_ of 0.003. Strains grown under InSPI2 conditions were washed 3 times in InSPI2 PCN medium and diluted in fresh PCN to a normalised OD_600_ of 0.03. Growth of each strain was assayed in a 96-well plate in a final volume of 200 μL at 37°C, with orbital shaking. Optical density was measured every 10 minutes throughout growth. Cell doubling times were calculated from the optical density measurements taken during exponential growth using www.doubling-time.com. Each strain was assayed in triplicate on 3 independent occasions.

### Ectopic expression analysis

For ectopic expression of proteins from the arabinose-inducible P_BAD_ promoter, overnight cultures of strains carrying the pBAD plasmid containing the cloned gene and an empty pBAD vector were set up as previously described. Overnight cultures were diluted in 25 mL of the appropriate medium in a 250 mL flask and grown to the desired OD_600_. Cultures were split into two 250 mL flasks and L-arabinose was added at a final concentration of 0.2% to one flask, to induce expression from the P_BAD_ promoter, while no arabinose was added to the second culture. Induction proceeded for the indicated length of time and cells were harvested for analysis.

### Genetic manipulation of *S*. Typhimurium strain 4/74

Gene deletion mutants were generated as previously described [89]. Genes were tagged in their chromosomal location using an adapted λ Red recombineering protocol as previously described [90]. Mutations or FLAG tags were moved into clean genetic backgrounds by P22 transduction and confirmed by PCR and DNA sequencing (Source Biosciences, Dublin). To ensure antibiotic resistance cassettes would not affect transcription of downstream genes, the resistance genes were removed from mutant strains using the pCP20 plasmid [89, 91]. Antibiotic resistant mutants or tagged strains were grown to mid-log phase and cells were transformed with the FLP recombinase-harbouring pCP20 plasmid. Cells were recovered for 1 hour at 30°C with aeration and plated on ampicillin or chloramphenicol plates. Transformants were passaged at 37°C without antibiotics to cure the strain of the pCP20 plasmid. Loss of the antibiotic resistance cassette and the pCP20 plasmid were screened for using appropriate antibiotic selection plates and incubation at permissive temperatures.

Sequence and ligation independent cloning (SLIC) was carried out with modifications [92]. Wild-type chromosomal DNA and plasmid DNA were used as templates to amplify the insert DNA and vector backbone respectively. One μg of DpnI-digested vector backbone and 1μg of insert DNA were treated with 5 units of T4 DNA polymerase in the absence of deoxynucleotide triphosphates to generate single strand overhangs. T4 DNA polymerase treatment proceeded at 23°C for 30 minutes in the presence of 5 mM DTT, 200 mM Urea, 1× BSA, and 1× reaction buffer (67 mM Tris-HCl pH8.8, 6.6 mM MgCl_2_, 1 mM DTT, 16.8 mM (NH_4_)_2_SO_4_). The reaction was stopped by addition of 25 mM EDTA and incubation at 75°C for 20 minutes. 100 ng of T4 DNA polymerase treated vector was mixed with an equal amount of T4 DNA polymerase treated “insert DNA” in a final volume of 10 μL. Samples were incubated at 65°C for 10 minutes followed by “Touch-down” annealing, during which the incubation temperature was reduced from 65°C to 25°C in 1°C decrements and samples were held for 1 minute at each temperature. Annealed vector and insert mixtures were then transformed into chemically competent *E*. *coli* TOP10 cells. Positive clones were selected for using appropriate antibiotic selection plates and overnight incubation at 37°C. Clones were screened by colony PCR using plasmid-specific and gene-specific primers.

All constructs, gene deletions, and gene tags were confirmed by PCR and sequenced by DNA sequencing.

### Isolation of total RNA from *S*. Typhimurium 4/74

RNA was extracted from S. Typhimurium cells grown to a defined OD_600_ (Table 2). Total RNA was isolated from wild-type and mutant strains using TRIzol, as previously described [93]. Briefly, 5 OD_600_ units were removed from the culture and cellular transcription was stopped using 0.4× culture volume of a 5% phenol 95% ethanol “stop” solution. RNA was stabilised on ice, in stop solution, for at least 30 minutes before cells were harvested at 4,000 rpm for 10 minutes at 4°C. Pellets were re-suspended in 1 mL of TRIzol Reagent. 400 μL of chloroform was added and the samples were immediately and thoroughly mixed by inversion. Samples were moved to a Phase-lock tube (5 Prime) and the aqueous and organic phases were separated by centrifugation at 13,000 rpm for 15 minutes at room temperature. RNA was precipitated from the aqueous phase using isopropanol for 30 minutes at room temperature followed by centrifugation at 13,000 rpm for 30 minutes at room temperature. The RNA pellet was rinsed with 70% ethanol followed by centrifugation at 13,000 rpm for 10 minutes at room temperature. The RNA pellet was air-dried for 15 minutes and re-suspended in DEPC-treated water at 65°C with shaking at 900 rpm on a Thriller thermoshaker (Peqlab) for 5 minutes with occasional vortexing. RNA was kept on ice whenever possible and RNA was stored at -80°C. RNA quality was assessed using a 2100 Bioanalyser (Agilent). RNA to be used for cDNA library preparations was treated with 10 units of DNase I to remove any DNA present in the sample and samples were purified by phenol-chloroform extraction.

### Northern blotting

*In vitro* transcription by T7 RNA polymerase was used to generate Dig-labelled riboprobes (Dig Northern Starter kit, Roche). Total RNA was electrophoresed through an 8.3 M Urea, 1× TBE, 7% polyacrylamide gel. RNA was transferred to a positively charged nylon membrane using the Biometra Fastblot B43 semi-dry blotting apparatus at a constant amplitude of 125 mA for 30 minutes at 4°C. RNA was UV-crosslinked to the membrane at 120 mJ for 2 minutes. The membrane was equilibrated in hybridisation buffer for 1 hour at 62°C in pre-warmed DIG Easy Hyb solution in a rotating hybridisation oven. Boiled riboprobe was added to the pre-hybridising solution and hybridisation proceeded at 62°C overnight. The membrane was washed twice for a total of 10 minutes in pre-heated (62°C) stringency wash buffer 1 (2× saline-sodium citrate (SSC) buffer, 0.1% SDS) at room temperature with rocking on a see-saw rocker (Stuart), followed by 2 washes for a total of 30 minutes with room temperature stringency wash buffer 2 (0.5× SSC buffer, 0.1% SDS) with rocking at room temperature. Unspecific sites on the membrane were blocked using 1× casein-based blocking solution, diluted in maleic acid buffer (0.1 M maleic acid, 0.15 M NaCl adjusted to pH 7.5 using NaOH pellets) for 30 minutes at room temperature with rocking. Alkaline phosphatase conjugated polyclonal anti-digoxigenin Fab-fragment was diluted 1:10,000 in 1× blocking buffer and immunological detection of the membrane proceeded for 30 minutes at room temperature with rocking. The membrane was then washed twice for a total of 30 minutes in wash buffer (maleic acid buffer, 0.3% Tween-20). The membrane was incubated for 5 minutes in detection buffer (0.1 M Tris-HCl, 0.1 M NaCl, pH 9.5) and CDP-star was used as the chemiluminescent substrate. Enzymatic de-phosphorylation of CDP-star by alkaline phosphatase was then visualised using an ImageQuant LAS4000 Imager. To determine if RNA samples were equally loaded on the gel, membranes were stripped and re-probed for the 5S ribosomal RNA.

### RNA adapter-based cDNA library construction and RNA-seq

Strand-specific cDNA library preparation and high throughput cDNA sequencing (RNA-seq) of wild-type 4/74 and isogenic mutants was performed on DNase I-digested total RNA by Vertis Biotechnologie AG (Freising, Germany). No depletion or enrichment methods were used. RNA was fragmented by ultrasound. The 5’ end of each fragment was de-phosphorylated using tobacco acid pyrophosphatase (TAP) and a re-phosphorylated using polynucleotide kinase (PNK) for 5’ mono-phosphorylation. Poly(A)-tails were added to each fragment by poly(A) polymerase and an RNA adaptor, containing a 6–10 nucleotide bar-code, was ligated to the 5’-phosphate of each RNA fragment. First strand cDNA synthesis was performed using oligo (dT) priming and Moloney murine leukaemia virus reverse transcriptase (M-MLV RT). The resulting cDNA was amplified by PCR to approximately 20–30 ng/μL using a high fidelity DNA polymerase. cDNA was purified using the Agencourt AMPure XP kit (Beckman Coulter Genomics) and analysed by capillary electrophoresis. cDNA was sequenced on an Illumina HiSeq 2000 platform. Mutants were always sequenced in the same lane as their wild-type comparator.

### Mapping of RNA-seq data and differential expression analysis

Sequence reads obtained from RNA-seq experiments were mapped to the 4/74 reference genome using the Segemehl mapping software [94]. To map reads which contained poor quality bases at the 3’ end, we used an iterative trimming approach in which nucleotides were truncated in a stepwise manner from the 3’ end until the reads mapped uniquely or until the read length fell below 20 bases. Reads that did not map uniquely to a single chromosomal location were discarded. Data were normalised using the transcripts per million (TPM) method [95, 96]. A TPM value of 10 was used as the threshold for gene expression based on TPM values of indicator genes which were previously shown not to be expressed under a particular condition [9]. Differential expression of genes between WT and isogenic mutants was calculated from TPM values. Two independent biological replicates of wild-type RNA from ESP, LSP and InSPI2 cultures were used in independent RNA-seq runs. Correlative analysis was used to demonstrate the reproducibility of the RNA extraction, cDNA library preparation and RNA-seq methods (S4 Table). During downstream analyses, independently extracted RNA was used to confirm RNA-seq-based findings by northern blot or quantitative PCR. For visualization of sequence reads in IGB and JBrowse, the read depth was adjusted in relation to the cDNA library with the lowest number of reads. This was achieved by dividing the read coverage at each genomic position by the library size and multiplying it by the size of the smallest library (Δ*ompR/envZ*).

### Chromatin immunoprecipitation (ChIP)

ChIP was carried out as previously described [71]. Briefly, protein-DNA complexes were cross-linked by adding formaldehyde to a final concentration of 1% in a drop-wise manner with gentle stirring at room temperature for 30 minutes. Cross-linking reactions were quenched by the addition of ice-cold glycine to a final concentration of 0.125 M for 5 minutes with gentle stirring at room temperature. The cross-linked cells were harvested by centrifugation at 4°C at 4,000 rpm for 8 minutes and were re-suspended in 600 μL of lysis buffer (50 mM Tris-HCl pH 8.1, 10 mM EDTA, 1% SDS, 1× protease inhibitor tablet stock) and incubated on ice for 10 minutes. 1.4 mL dilution buffer (20 mM Tris-HCl pH 8.1, 150 mM NaCl, 2 mM EDTA, 1% Trition X-100, 0.01% SDS, 1× protease inhibitor tablet stock) was added and the chromatin was sonicated on ice to reduce the average DNA fragment length to approximately 500 bp using an MSE Soniprep sonicator (Sanyo). The chromatin was pre-cleared by adding 50 μg of non-species specific IgG (normal rabbit IgG, Millipore). The chromatin was incubated for 1 hour at 4°C on a rotating wheel and 100 μL of protein G-agarose bead suspension was added. The chromatin was incubated for a further 3 hours at 4°C with rotation. Beads were pelleted at 4,000 rpm for 4 minutes at 4°C. 200 μL of pre-cleared chromatin was removed and stored at -20°C as “Input” DNA for downstream analysis. The remainder of the pre-cleared chromatin was used to set up Immunoprecipitation (IP) reactions. A “mock” IP reaction containing 1350 μL of chromatin and 10 μg of species specific IgG (normal mouse IgG, Millipore) was set up to measure background levels of DNA binding to antibodies and beads. Experimental IP reactions contained 1350 μL of chromatin and 10 μg of monoclonal mouse anti-FLAG M2 antibody (Sigma). IP reactions were incubated overnight at 4°C on a rotating wheel. 50 μL of protein G-agarose bead suspension was added to each IP sample and incubation continued for 3 hours at 4°C on a rotating wheel. The beads containing the bound antibody-protein-DNA complexes were carefully washed. Antibody-protein-DNA complexes were eluted from the beads at room temperature by adding 225 μL of elution buffer (100 mM NaHCO_3_, 1% SDS) followed by vortexing and pelleting of the beads twice. Both eluates were combined in the same tube. Input and IP samples were treated with 5 ng/μL RNase A and 0.3 M NaCl and incubated at 65°C for at least 6 hours or overnight. Protein-DNA cross-links were disrupted by treating Input and IP samples with 9 μg of proteinase K at 45°C for at least 3 hours or overnight. DNA was extracted from Input and IP samples by standard phenol-chloroform extraction followed by ethanol precipitation with yeast tRNA and glycogen as co-precipitants. DNA was re-suspended in nuclease free water at 37°C with shaking at 900 rpm for 1 hour. Input and IP DNA was analysed by quantitative PCR and quantified relative to a standard curve of chromosomal DNA. The quantity of immunoprecipitated DNA is relative to specific protein binding in that region and was calculated as a fraction of the starting amount of DNA (Input). The mock immunoprecipitate and experimental immunoprecipitate were compared to a control region which was negative for specific transcription factor binding.

### Assignment of fitness scores for HilD and SsrB-controlled genes

Published data from two studies involving high-throughput analysis of mutant pools during infection showed the virulence-associated roles played by HilD and SsrB-controlled genes. The scoring methods used by the authors of each publication were applied independently to the relevant dataset. High-throughput sequencing of insertion sites of pools of *S*. Typhimurium transposon mutants was used to compare the ratio of input to output reads to determine relative fitness, following oral infections of chickens, pig or calves. A negative or positive fitness score indicates attenuation (blue) or amplification (orange) of fitness, respectively, as a result of the transposon insertion. If no output reads were identified for a particular insertion an arbitrary negative fitness score of -15 was assigned [41]. Two *S*. Typhimurium single gene deletion libraries were used to infect BALB/c mice via intraperitoneal infection, and were isolated from the spleen and liver; DNA microarrays were used to compare the ratio of input to output reads to determine fitness. A negative or positive fitness score indicates attenuation (blue) or amplification (orange) of fitness, respectively, as a result of the gene deletion [42]. In cases where different fitness scores were assigned to individual mutants isolated from different sites of the host, the most negative or most positive score was chosen.

### Hfq enrichment

Enrichment for binding by Hfq was determined, as previously described [9], using published Hfq co-immunoprecipitation datasets [51, 78]. Briefly, a 5-fold enrichment factor of Hfq immunoprecipitation over a mock immunoprecipitation was used to determine if sRNAs were strongly associated with Hfq.

### Motif analysis of the Fur recognition site

A position-specific scoring matrix (PSSM) was generated using alignment of the homologous *Salmonella* sequences of 15 published Fur binding sites from *E*. *coli* (available at http://arep.med.harvard.edu/ecoli_matrices/), by assigning a score to each possible base at each position within the binding site and normalising to the average G/C content in the *S*. Typhimurium chromosome. The PSSM was used to scan for direct Fur binding, in the 100 bp upstream of the 30 sRNA genes which were up-regulated in the Δ*fur* mutant, using pattern searching software (available from rsat.ulb.ac.be), as 100 bp was the maximum distance reported for Fur binding upstream of the published Fur-regulated genes used for matrix assembly. The published Fur binding sequences were scanned with the same PSSM to establish a minimum threshold weighted score, below which predicted motifs were considered to be false-positives. Because the lowest weighted score of a published Fur binding site was 7, and a number of poorly conserved motifs had a score of approximately 7, we chose the more conservative threshold score of 10. Only motifs with a weighted score >10 were designated as putative Fur-binding sites.

### Motif analysis of the SsrB recognition site

Previously published ChIP-chip analysis of the SsrB regulon identified an 18 bp palindromic sequence with an internal 7-4-7 organisation for SsrB recognition [47]. A position-specific scoring matrix (PSSM) was generated from an alignment of these previously identified SsrB-bound sites by assigning a score to each possible base at each position within the binding site and normalising to the average G/C content in the *S*. Typhimurium chromosome. The PSSM was used to scan approximately 500 bp upstream of the sRNAs which were down-regulated in the absence of SsrB, using pattern searching software available from rsat.ulb.ac.be. A score of 10 was used as the minimum threshold score for an SsrB recognition motif, based on the scores of defined SsrB bound sites scanned with the same PSSM.

### Phylogenetic analysis

Investigation of the conservation of 15 transcription factors, TCS response regulators and Sigma factors was determined in 29 enterobacterial genomes using GLSEARCH. 1.00 indicates 100% sequence identity. *Salmonella*-specific regulators were defined as those with >90% sequence identity within the *Salmonella* genus and <70% sequence identity within other members of the *Enterobacteriaceae* family.

### Accession numbers

The RNA-seq data generated from this study are deposited at NCBI GEO under the accession numbers GSM2091439 to GSM2091466, and can be accessed at http://www.ncbi.nlm.nih.gov/geo/query/acc.cgi?acc=GSE79314.

## Supporting Information

S1 Fig Schematic showing a simplified *S*. Typhimurium regulatory network. Schematic showing the key regulatory interactions required for *S*. Typhimurium pathogenicity and the control of sRNA expression. Transcriptional activation is represented by full red arrows, transcriptional repression is represented by full blue T-bars, post-transcriptional activation is represented by dotted red arrows, post-transcriptional repression is represented by dotted blue T-bars. Extracellular environmental signals are represented by wavy black arrows. Membrane-bound sensor kinases, outer membrane porins and transporter systems are shown within the cellular membrane. Cell surface appendages, such as Type III Secretion Systems, flagella, fimbriae and curli fibres are indicated outside the cell. In the case of indirect regulatory interactions, mediators of the interaction are indicated in grey boxes. The schematic is based on a number of publications, including [4, 20, 24, 29, 39, 56, 97] and others. Some regulatory interactions have been omitted for ease of viewing.
(TIF)

S2 Fig Overlap between the SsrA, SsrB and SsrAB regulons. Venn diagram showing the high level of similarity between the differentially-expressed genes in the single Δ*ssrA*, Δ*ssrB* and the double Δ*ssrAB* mutants compared to the wild-type strain grown under SPI2-inducing conditions. Individual gene lists are available in S3 Table.
(TIF)

S3 Fig Chromosomal location and Hfq-enrichment of differentially-expressed sRNAs. Stacked bar chart showing the number of differentially-expressed sRNAs (>3-fold change in expression in panel of regulatory mutants, compared to wild-type) in terms of their chromosomal location relative to nearby coding genes and in the context of enrichment for Hfq, as determined by Chao et al (2012) using Hfq co-immunoprecipitation [51]. The height of each bar reflects the number of sRNAs in each category.
(TIF)

S4 Fig Variation in size of regulons comprising sRNAs and CDS. (A) Stacked bar chart showing the numbers of differentially-expressed sRNAs and (B) the numbers of differentially-expressed CDS in the panel of regulatory mutants. Red indicates the number of genes which show decreased expression (activated by regulatory system) in the mutant strain. Blue indicates the number of genes which show increased expression (repressed by regulatory system) in the mutant strain. In each case the comparator is the wild-type strain grown under the same environmental condition.
(TIF)

S5 Fig Predicted binding of Fur within sRNA promoter regions. (A) Motif consensus logo for Fur recognition motif was generated with software available from http://weblogo.berkeley.edu [98], using published Fur binding sites (http://arep.med.harvard.edu/ecoli_matrices/). (B) Consensus sequence for Fur recognition aligned with putative Fur recognition sites within Fur-regulated sRNA promoters. (C) Genetic organisation of the STnc4000 and STnc3250 promoters demonstrating locations of the putative Fur recognition sites.
(TIF)

S6 Fig Predicted RpoS-dependent promoters. Multiple alignment of the promoters of RpoS-dependent sRNAs that contain conserved hallmarks of RpoS recognition [66]. Some promoter features that favour RpoS recognition are indicated in red above the alignment. Within the sequence alignment: Red >75% nucleotide sequence identity; Blue >35% <75% nucleotide sequence identity; Black <35% nucleotide sequence identity between aligned promoter sequences.
(TIF)

S7 Fig The SPI1-like sRNA STnc520 is directly regulated by the SPI1-encoded transcription factor SprB. (A) Northern blot showing expression of STnc520 in the wild-type *Salmonella* Typhimurium 4/74 and isogenic mutants of SPI1-encoded or SPI1-associated transcription factors. 5S RNA was probed as a loading control. (B) Chromatin immunoprecipitation (ChIP) followed by qPCR demonstrates SprB binding to the STnc520 promoter. There is strong enrichment (approximately 14-fold) of the STnc520 promoter region in the experimental (FLAG) ChIP DNA, compared to the background “mock” ChIP DNA. The negative control gene, *hemX*, displayed little enrichment in the experimental ChIP DNA sample, compared to the mock DNA. Error bars are based on the standard deviation from 2 independent biological replicates.
(TIF)

S8 Fig Predicted binding of SsrB within the STnc3020 promoter region. (A) Motif consensus logo for SsrB recognition motif was generated using Weblogo software (http://weblogo.berkeley.edu) [98] using SsrB bound sites determined by ChIP-chip analysis [47]. (B) Consensus sequence for SsrB recognition aligned with putative SsrB recognition site within the STnc3020 promoter. (C) Genetic organisation of the STnc3020 promoter demonstrating the location of the predicted SsrB recognition site.
(TIF)

S1 Table Dataset 1: Doubling times of wild-type and mutant strains in relevant growth media. (XLSX)

S2 Table Dataset 2: RNA-seq statistics for 3 Illumina HiSeq experiments. (XLSX)

S3 Table Dataset 3–22: Differentially-expressed CDS (excluding deleted CDS for each mutant) and sRNA genes in all mutant strains. (XLSX)

S4 Table Dataset 23: Pearson correlation coefficients showing the high reproducibility of RNA-seq data between independent biological replicates. (XLSX)

S5 Table Dataset 24: Phylogenetic analysis of 15 transcription factors. Dataset 25: Phylogenetic analysis of 13 SPI1 and SPI2-like sRNAs. Dataset 26: Phylogenetic analysis of 30 Hfq-associated sRNAs.
(XLSX)

S6 Table Dataset 27: Regulatory inputs to differentially-expressed sRNAs. Dataset 28: chromosomal location of differentially-expressed sRNAS. Dataset 29: 3’UTR sRNAs. Dataset 30: Hfq associated sRNAs.
(XLSX)

S1 Text Discussion of the mutations used in this study that showed minor polar effects and the overlaps between virulence gene expression profiles from this study with the literature. (DOCX)
