# Supplementary figures and images for: The Impact of 18 Ancestral and Horizontally-Acquired Regulatory Proteins upon the Transcriptome and sRNA Landscape of Salmonella enterica serovar Typhimurium
Source: PLoS Genet. 2016 Aug 26;12(8):e1006258. doi: 10.1371/journal.pgen.1006258 (PMC5001712; doi:10.1371/journal.pgen.1006258)

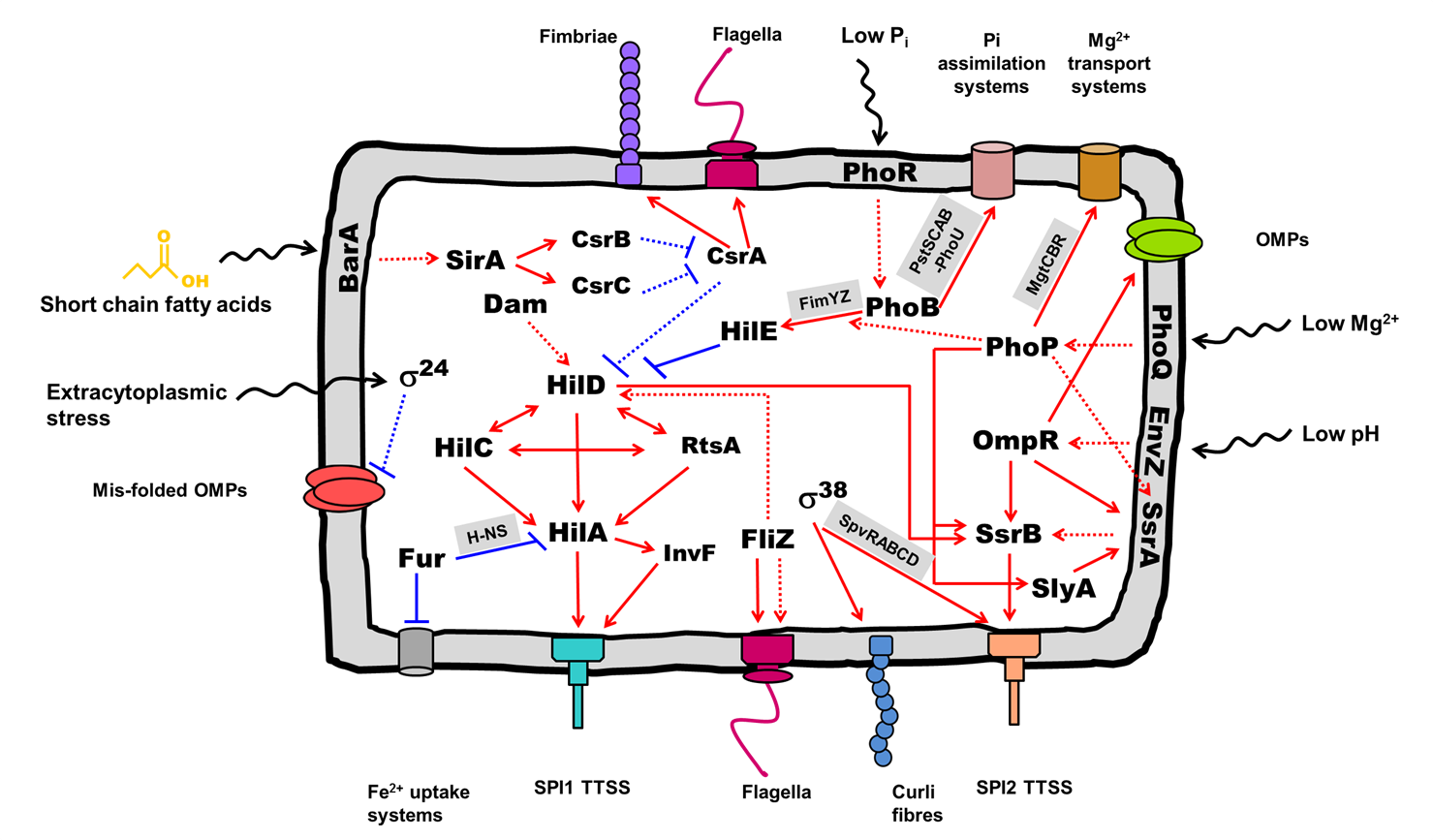

Supplement: S1 Fig — Schematic showing the key regulatory interactions required for S. Typhimurium pathogenicity and the control of sRNA expression. Transcriptional activation is represented by full red arrows, transcriptional repression is represented by full blue T-bars, post-transcriptional activation is represented by dotted red arrows, post-transcriptional repression is represented by dotted blue T-bars. Extracellular environmental signals are represented by wavy black arrows. Membrane-bound sensor kinases, outer membrane porins and transporter systems are shown within the cellular membrane. Cell surface appendages, such as Type III Secretion Systems, flagella, fimbriae and curli fibres are indicated outside the cell. In the case of indirect regulatory interactions, mediators of the interaction are indicated in grey boxes. The schematic is based on a number of publications, including [4, 20, 24, 29, 39, 56, 97] and others. Some regulatory interactions have been omitted for ease of viewing. (TIF) [file pgen.1006258.s001.tif]

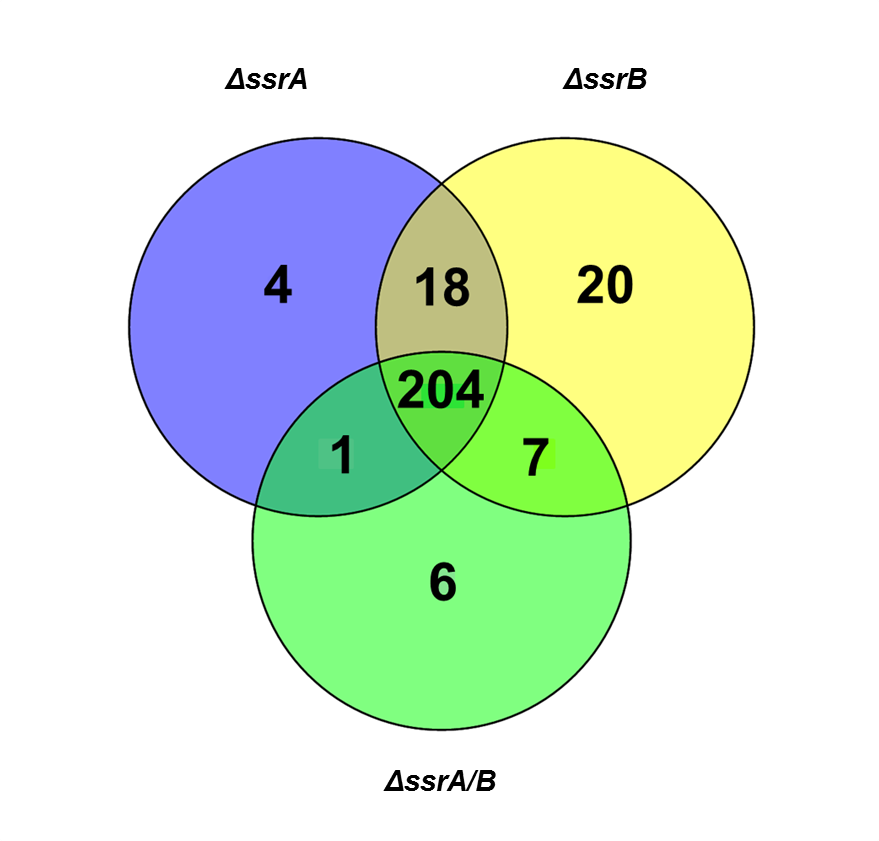

Supplement: S2 Fig — Venn diagram showing the high level of similarity between the differentially-expressed genes in the single ΔssrA, ΔssrB and the double ΔssrAB mutants compared to the wild-type strain grown under SPI2-inducing conditions. Individual gene lists are available in S3 Table. (TIF) [file pgen.1006258.s002.tif]

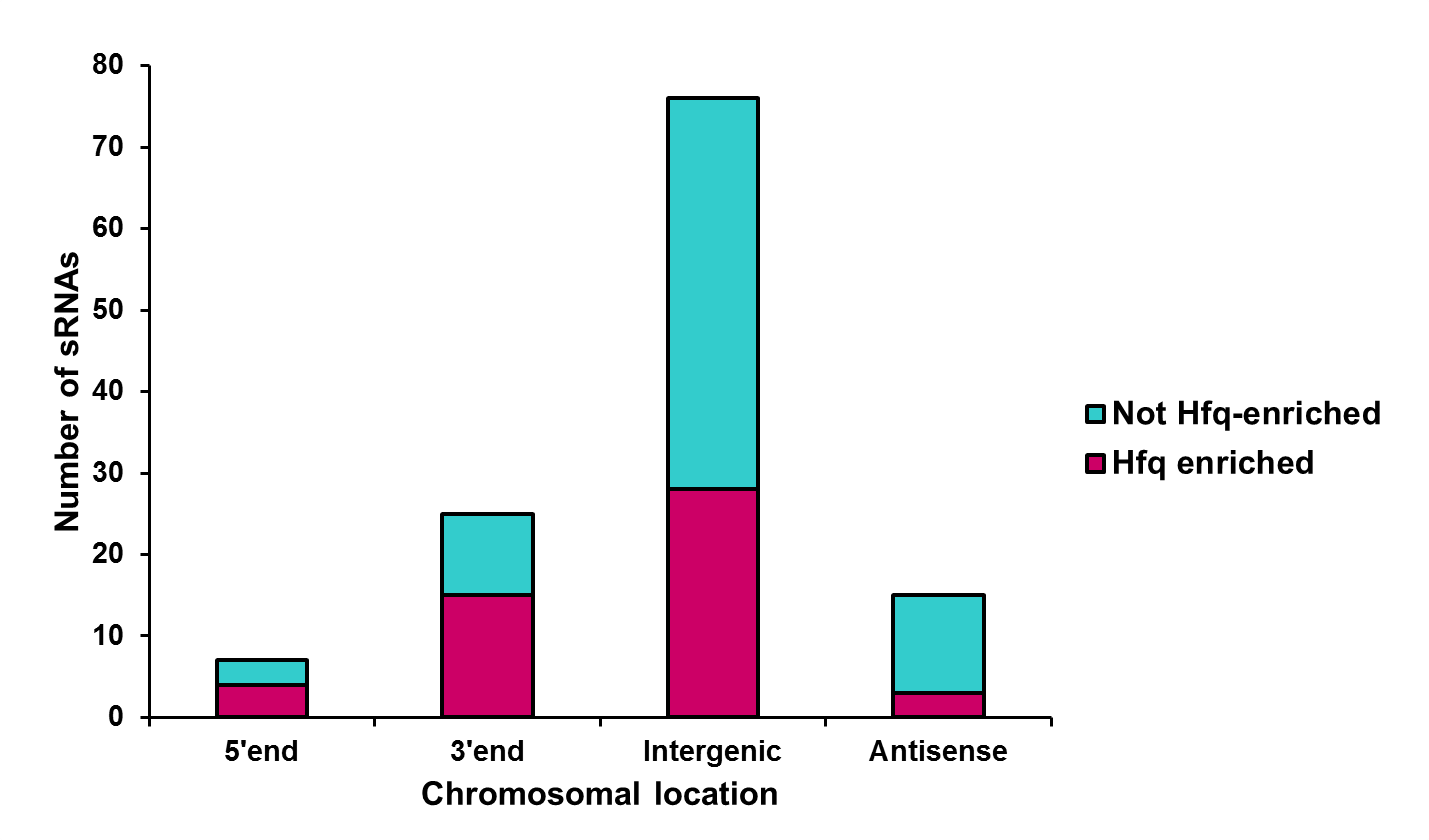

Supplement: S3 Fig — Stacked bar chart showing the number of differentially-expressed sRNAs (>3-fold change in expression in panel of regulatory mutants, compared to wild-type) in terms of their chromosomal location relative to nearby coding genes and in the context of enrichment for Hfq, as determined by Chao et al (2012) using Hfq co-immunoprecipitation [51]. The height of each bar reflects the number of sRNAs in each category. (TIF) [file pgen.1006258.s003.tif]

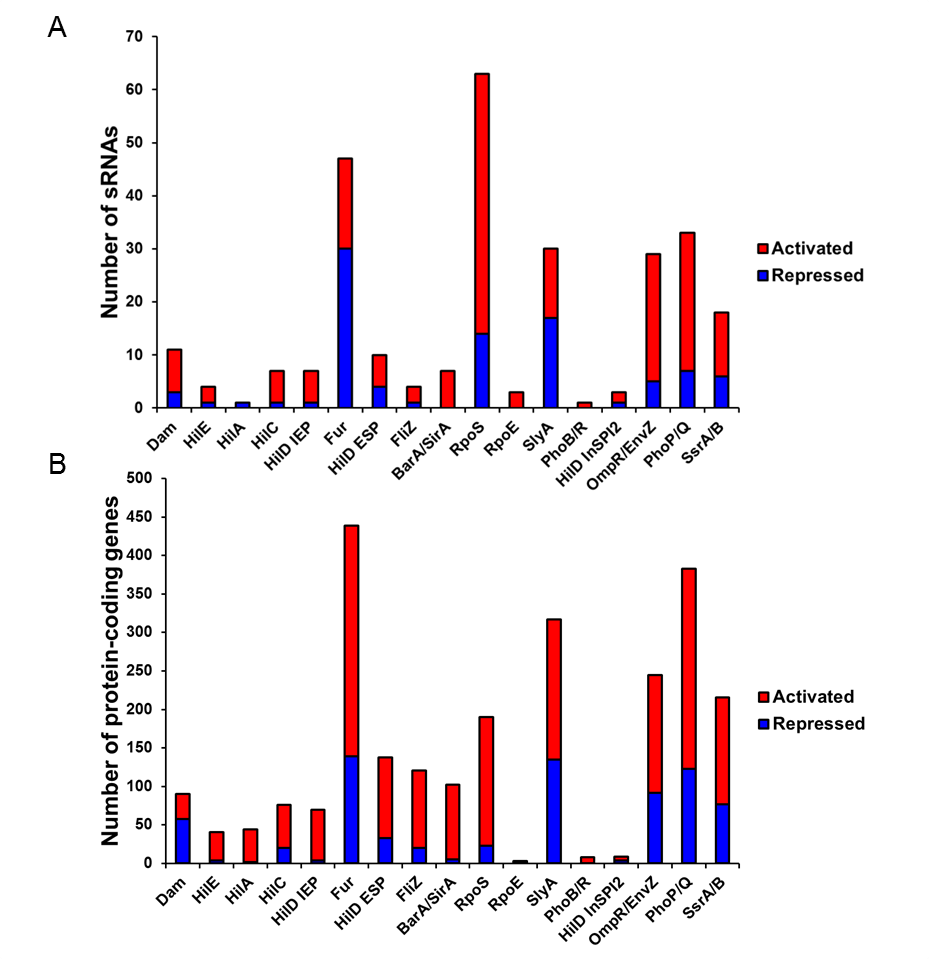

Supplement: S4 Fig — (A) Stacked bar chart showing the numbers of differentially-expressed sRNAs and (B) the numbers of differentially-expressed CDS in the panel of regulatory mutants. Red indicates the number of genes which show decreased expression (activated by regulatory system) in the mutant strain. Blue indicates the number of genes which show increased expression (repressed by regulatory system) in the mutant strain. In each case the comparator is the wild-type strain grown under the same environmental condition. (TIF) [file pgen.1006258.s004.tif]

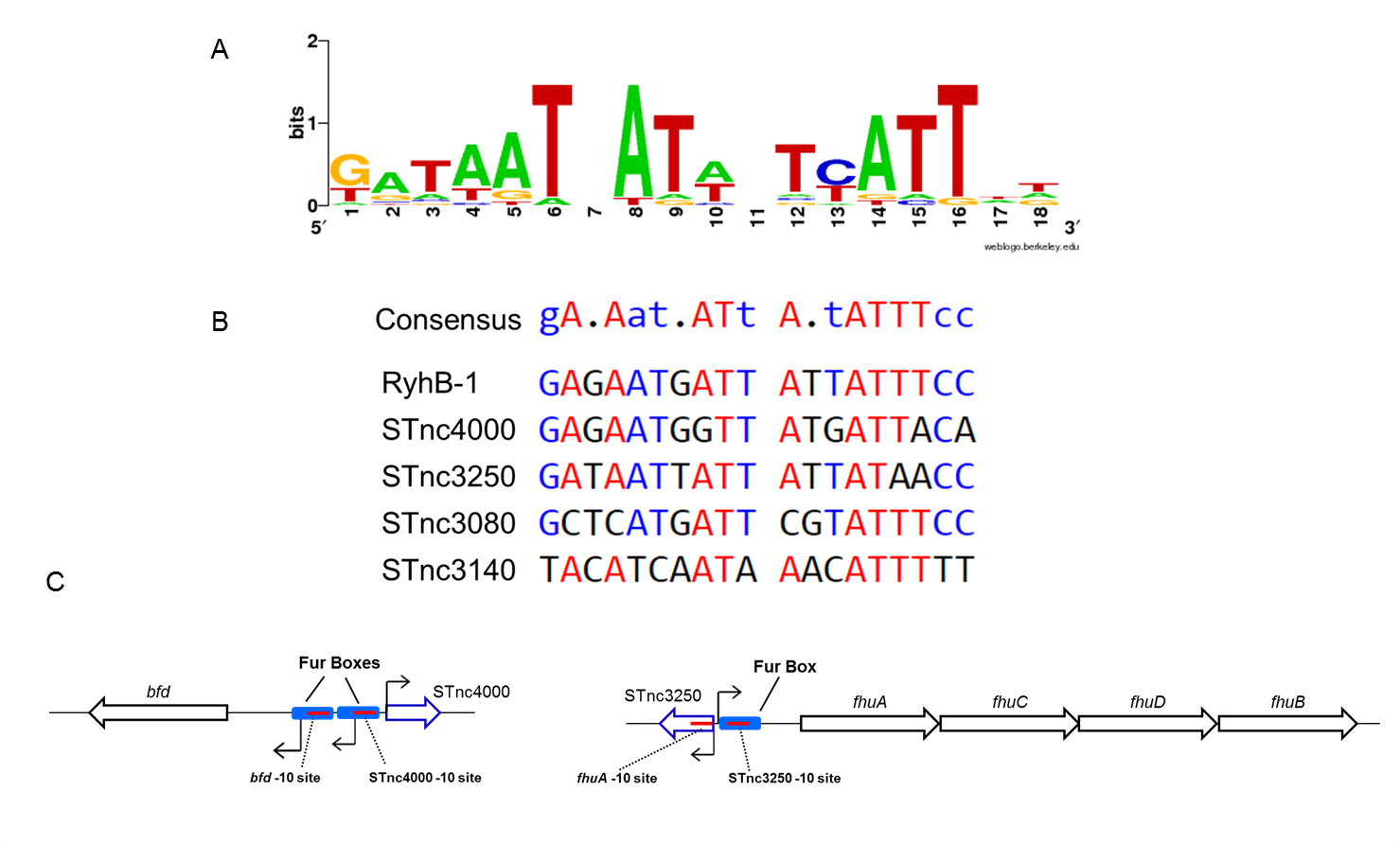

Supplement: S5 Fig — (A) Motif consensus logo for Fur recognition motif was generated with software available from http://weblogo.berkeley.edu [98], using published Fur binding sites (http://arep.med.harvard.edu/ecoli_matrices/). (B) Consensus sequence for Fur recognition aligned with putative Fur recognition sites within Fur-regulated sRNA promoters. (C) Genetic organisation of the STnc4000 and STnc3250 promoters demonstrating locations of the putative Fur recognition sites. (TIF) [file pgen.1006258.s005.tif]

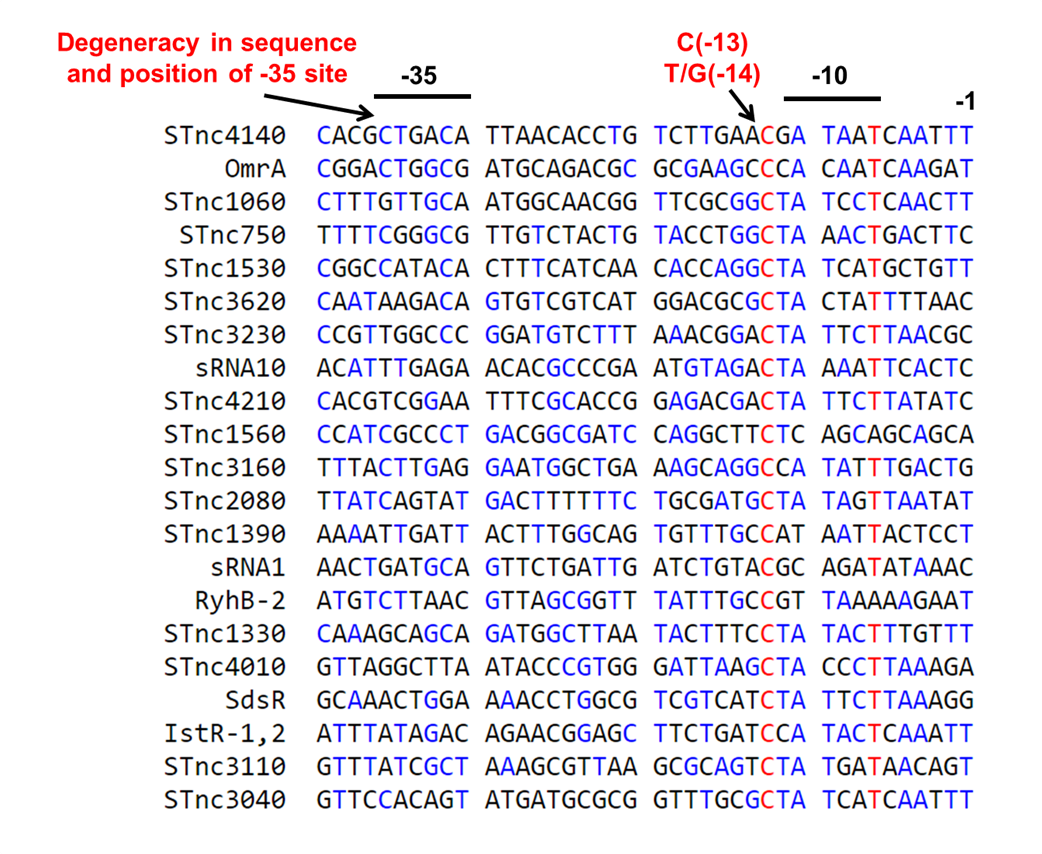

Supplement: S6 Fig — Multiple alignment of the promoters of RpoS-dependent sRNAs that contain conserved hallmarks of RpoS recognition [66]. Some promoter features that favour RpoS recognition are indicated in red above the alignment. Within the sequence alignment: Red >75% nucleotide sequence identity; Blue >35% <75% nucleotide sequence identity; Black <35% nucleotide sequence identity between aligned promoter sequences. (TIF) [file pgen.1006258.s006.tif]

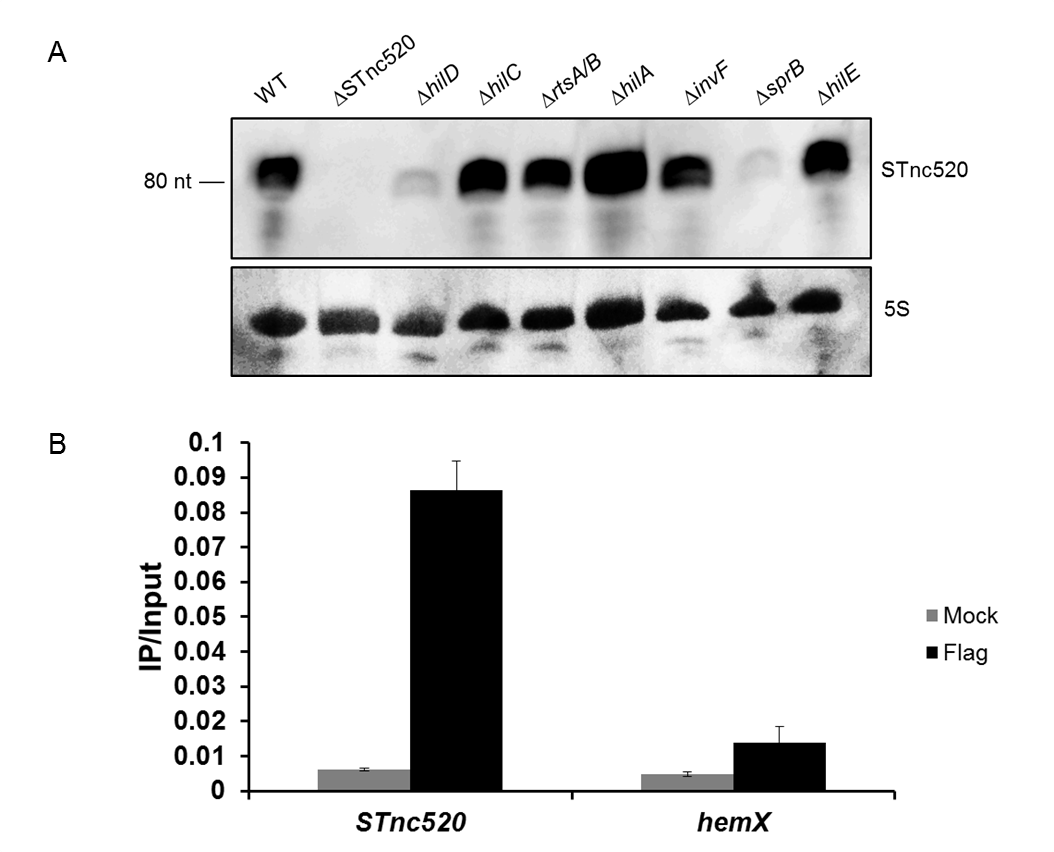

Supplement: S7 Fig — (A) Northern blot showing expression of STnc520 in the wild-type Salmonella Typhimurium 4/74 and isogenic mutants of SPI1-encoded or SPI1-associated transcription factors. 5S RNA was probed as a loading control. (B) Chromatin immunoprecipitation (ChIP) followed by qPCR demonstrates SprB binding to the STnc520 promoter. There is strong enrichment (approximately 14-fold) of the STnc520 promoter region in the experimental (FLAG) ChIP DNA, compared to the background “mock” ChIP DNA. The negative control gene, hemX, displayed little enrichment in the experimental ChIP DNA sample, compared to the mock DNA. Error bars are based on the standard deviation from 2 independent biological replicates. (TIF) [file pgen.1006258.s007.tif]

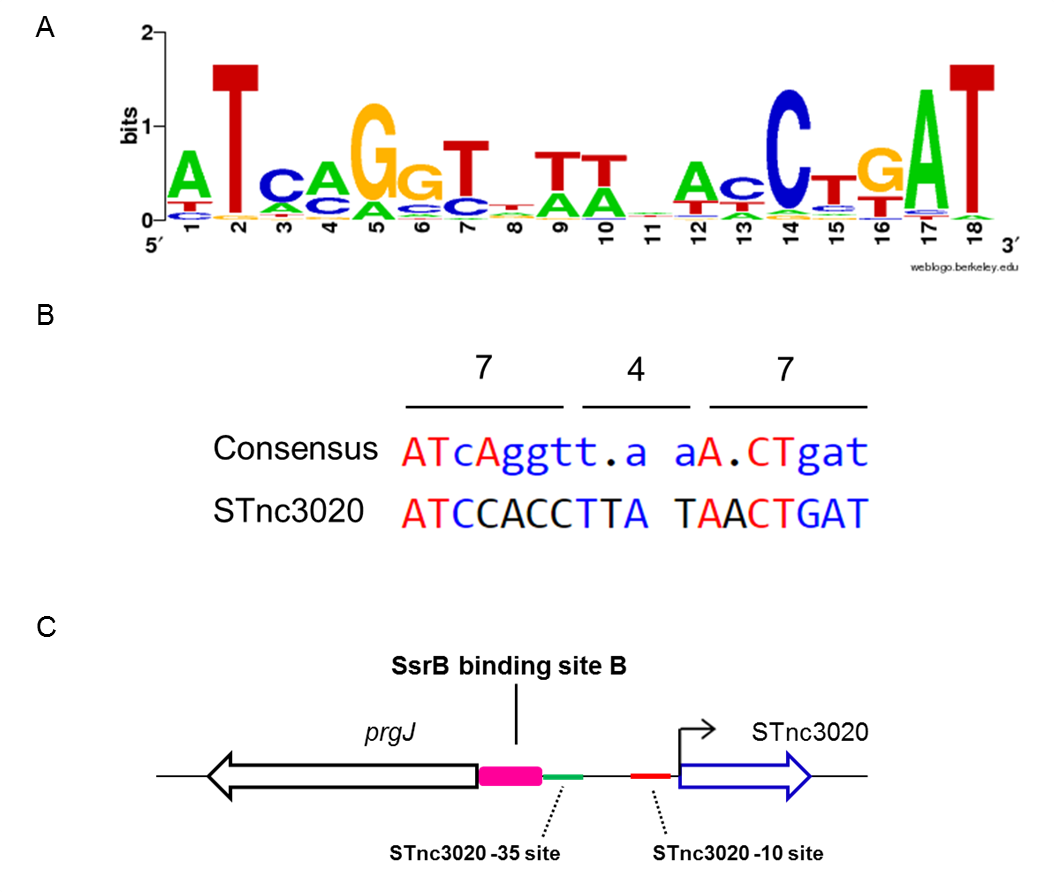

Supplement: S8 Fig — (A) Motif consensus logo for SsrB recognition motif was generated using Weblogo software (http://weblogo.berkeley.edu) [98] using SsrB bound sites determined by ChIP-chip analysis [47]. (B) Consensus sequence for SsrB recognition aligned with putative SsrB recognition site within the STnc3020 promoter. (C) Genetic organisation of the STnc3020 promoter demonstrating the location of the predicted SsrB recognition site. (TIF) [file pgen.1006258.s008.tif]
