# Supplementary material for: The Impact of 18 Ancestral and Horizontally-Acquired Regulatory Proteins upon the Transcriptome and sRNA Landscape of Salmonella enterica serovar Typhimurium
Source: PLoS Genet. 2016 Aug 26;12(8):e1006258. doi: 10.1371/journal.pgen.1006258 (PMC5001712; doi:10.1371/journal.pgen.1006258)
Supplement: S1 Text — (DOCX) [file pgen.1006258.s015.docx]

**Polarity effects of deletion mutations.**

The possibility that deletion mutants used in this study (Table 1) had polar effects on transcription was assessed by RNA-seq (Figure 1). The 3’ end of the *sirA* coding sequence overlaps with the TSS of the downstream *uvrC* gene (Fig 1A). To maintain an intact *uvrC* TSS, only 432 bp of the *sirA* gene were removed during mutant construction and the 3’ end of the *sirA* gene was not deleted. However, expression of the *uvrC* gene was reduced approximately 4-fold in the Δ*barA/sirA*-432 mutant. The reduction in expression of *uvrC* may be a result of direct or indirect regulation by the BarA/SirA two-component system. However, in *E. coli, uvrY* (*sirA*) and *uvrC* are expressed as an operon and the majority of *uvrC* expression is driven from the promoter upstream of *uvrY* [[1](#_ENREF_1)] and this is also likely to be the case for the *Salmonella uvrYC* genes. The *uvrC* gene encodes an endonuclease that is part of the UvrABC system which detects and aids in the repair of DNA damage by nucleotide excision. While there is a constitutive basal level of *uvrC* expression in *E. coli*, the gene is mostly repressed by the LexA repressor and becomes activated as part of the SOS response [[1](#_ENREF_1)]. The Δ*barA/sirA*-432 mutant and wild-type comparator were grown in rich media to early stationary phase without mutagens that cause DNA damage or induce the SOS response, so UvrC expression is not likely to be important under this condition.

Expression of *ssrA* was reduced approximately 4-fold in the Δ*ssrB* mutant (Fig 1B). This reduction in *ssrA* expression was likely to be a regulatory effect rather than a technical effect, as SsrB binds and activates expression from the *ssrA* promoter [[2](#_ENREF_2)]. Expression of the gene downstream of *ssrAB* locus, *orf242*, was also reduced approximately 4-fold in the absence of SsrA and SsrB (Fig 1B). *orf242* is transcribed from its own promoter and the reduction in *orf242* expression was likely to be regulatory as SsrB binds in the intergenic region upstream of *orf242* [[2](#_ENREF_2)].

*ycfD* encodes a gene of unknown function and is transcribed from the *phoP/Q* promoter (Fig 1B). Expression of *ycfD* was reduced approximately 5-fold as a result of deleting the *phoP* and *phoQ* genes. Transcription from the *phoP/Q* promoter is auto-regulated by PhoP, so a reduction in *ycfD* expression would be expected in the absence of PhoP. However, deletion of the *ycfD* gene does not seem to have a broad effect on cellular regulons as a kanamycin resistance cassette was previously introduced into this gene to mediate co-transduction of the constitutive *phoQ24* mutation into various genetic backgrounds by other laboratories [[3](#_ENREF_3)].

**Differential gene expression profiles that disagree with by the literature.**

The regulatory impact of individual transcription factors were assessed by comparing expression profiles of deletion mutants with published studies. Expression of *fljB* decreases approximately 3-fold in the absence of the BarA/SirA two component system, despite the moderate BarA/SirA-dependent increase in expression of genes encoding components of the flagellar biosynthesis pathway that we observed, and that has been previously reported [[4](#_ENREF_4)]. The decrease in *fljB* expression may reflect flagellar phase variation as *Salmonella* alternates between expression of the FljB and FliC flagellin subunits throughout growth [[5](#_ENREF_5)], regardless of expression of other flagellar regulons.

Phase variation may also explain the decrease in *fliC* expression in the absence of the Dam protein which conflicts with the previously reported repression of *fliC* expression by Dam [[6](#_ENREF_6)].

The expression of SPI1 genes increases in the absence of Dam in this study, unlike the previously reported Dam-mediated activation of SPI1 [[6](#_ENREF_6)]. We suggest these differences may arise from the differences in growth media used in both studies. Balbontín *et al* grew the wild-type and ∆*dam* mutant to mid-log phase in Luria-Bertani media, which contains NaCl at a concentration of 172 µM, while Lennox media contains less salt (86 µM NaCl). SPI1 gene expression is activated under conditions of high salt [[7](#_ENREF_7)], while SPI1 genes are not expressed or are expressed at low levels in wild-type *S.* Typhimurium under the conditions used in our study. We speculate that the reduced levels of NaCl used here affect Dam-mediated post-transcriptional regulation of *hilD* [[8](#_ENREF_8)].

1. Stark T, Moses RE. Interaction of the LexA repressor and the *uvrC* regulatory region. FEBS Lett. 1989;258(1):39-41. Epub 1989/11/20.

2. Tomljenovic-Berube AM, Mulder DT, Whiteside MD, Brinkman FS, Coombes BK. Identification of the regulatory logic controlling *Salmonella* pathoadaptation by the SsrA-SsrB two-component system. PLoS Genet. 2010;6(3):e1000875. Epub 2010/03/20.

3. Golubeva YA, Sadik AY, Ellermeier JR, Slauch JM. Integrating global regulatory input into the *Salmonella* pathogenicity island 1 type III secretion system. Genetics. 2012;190(1):79-90. Epub 2011/10/25.

4. Teplitski M, Goodier RI, Ahmer BM. Pathways leading from BarA/SirA to motility and virulence gene expression in *Salmonella*. J Bacteriol. 2003;185(24):7257-65. Epub 2003/12/04.

5. Silverman M, Simon M. Phase variation: genetic analysis of switching mutants. Cell. 1980;19(4):845-54. Epub 1980/04/01.

6. Balbontin R, Rowley G, Pucciarelli MG, Lopez-Garrido J, Wormstone Y, Lucchini S, et al. DNA adenine methylation regulates virulence gene expression in *Salmonella enterica* serovar Typhimurium. J Bacteriol. 2006;188(23):8160-8. Epub 2006/09/26.

7. Eichelberg K, Galan JE. Differential regulation of *Salmonella* typhimurium type III secreted proteins by pathogenicity island 1 (SPI-1)-encoded transcriptional activators InvF and hilA. Infect Immun. 1999;67(8):4099-105. Epub 1999/07/23.

8. Lopez-Garrido J, Casadesus J. Regulation of *Salmonella enterica* pathogenicity island 1 by DNA adenine methylation. Genetics. 2010;184(3):637-49. Epub 2009/12/17.
